# Supplementary material for: The Importance of Strain (Preorganization) in Beryllium Bonds
Source: Molecules. 2020 Dec 11;25(24):5876. doi: 10.3390/molecules25245876 (PMC7763456; doi:10.3390/molecules25245876)
Supplement: Supplementary file 1 [file molecules-25-05876-s001.pdf]

The importance of strain (preorganization) in beryllium bonds.

Ibon Alkorta,* José Elguero
Instituto de Química Médica (CSIC), Juan de la Cierva, 3, E-28006 Madrid, Spain.
ibon@iqm.csic.es

Josep M. Oliva-Enrich*
Instituto de Química-Física "Rocasolano" (CSIC), Serrano, 119, E-28006 Madrid, Spain
j.m.oliva@iqfr.csic.es

Manuel Yáñez,* Otilia Mó, M. Merced Montero
Departamento de Química, Facultad de Ciencias, Módulo 13, and Institute of Advanced Chemical Sciences (IadChem). Universidad Autónoma de Madrid, Campus de Excelencia UAM-CSIC, Cantoblanco, E-28049 Madrid, Spain.
manuel.yanez@uam.es

Index:

Pg. S2	Fig. S1 Molecular electrostatic potential (MESP) on the 0.001 au electron density. Regions with MESP >0.1 au are indicated with blue color and MESP < -0.015 au with red color. The location of the $V_{S,max}$ is indicated with a black sphere.
Pg. S3	Fig. S2. LUMO of the $B_9H_{11}C_2Be$ isomers.
Pg. S4	Table S1. Energy (a.u.) of the LUMO of the $B_9H_{11}C_2Be$ isomers.
Pg. S4	Fig. S3. Linear correlation between the $V_{s,max}$ of the MESP and the absolute value of the energy of the LUMO.
Pg. S5-31	Table S2. Molecular graphs and optimized Cartesian coordinates (Å) of the complexes.
Pg. S32	Table S3. Electron density (au) at the BCP between the Be atom and the Lewis base.
Pg. S33	Fig. S4. Electron density properties (ρ_{BCP} and $\nabla^2\rho_{BCP}$) at the intermolecular BCPs (au) vs. the interatomic distance (Å).
Pg. S34-35	Table S4. LMOEDA energy terms (kJ mol^{-1}).
Pg. S36	Fig. S5. Graphical representation of the LMOEDA energy terms (kJ mol^{-1})
Pg. S37	Fig. S6. Intermolecular distances in A complexes vs. B-D complexes (Å)
Pg. S38	Table S5. Dissociation Δ*G* energies (kJ mol^{-1})
Pg. S38	Fig. S7. Dissociation enthalpies of A complexes vs. B-D complexes (kJ mol^{-1})

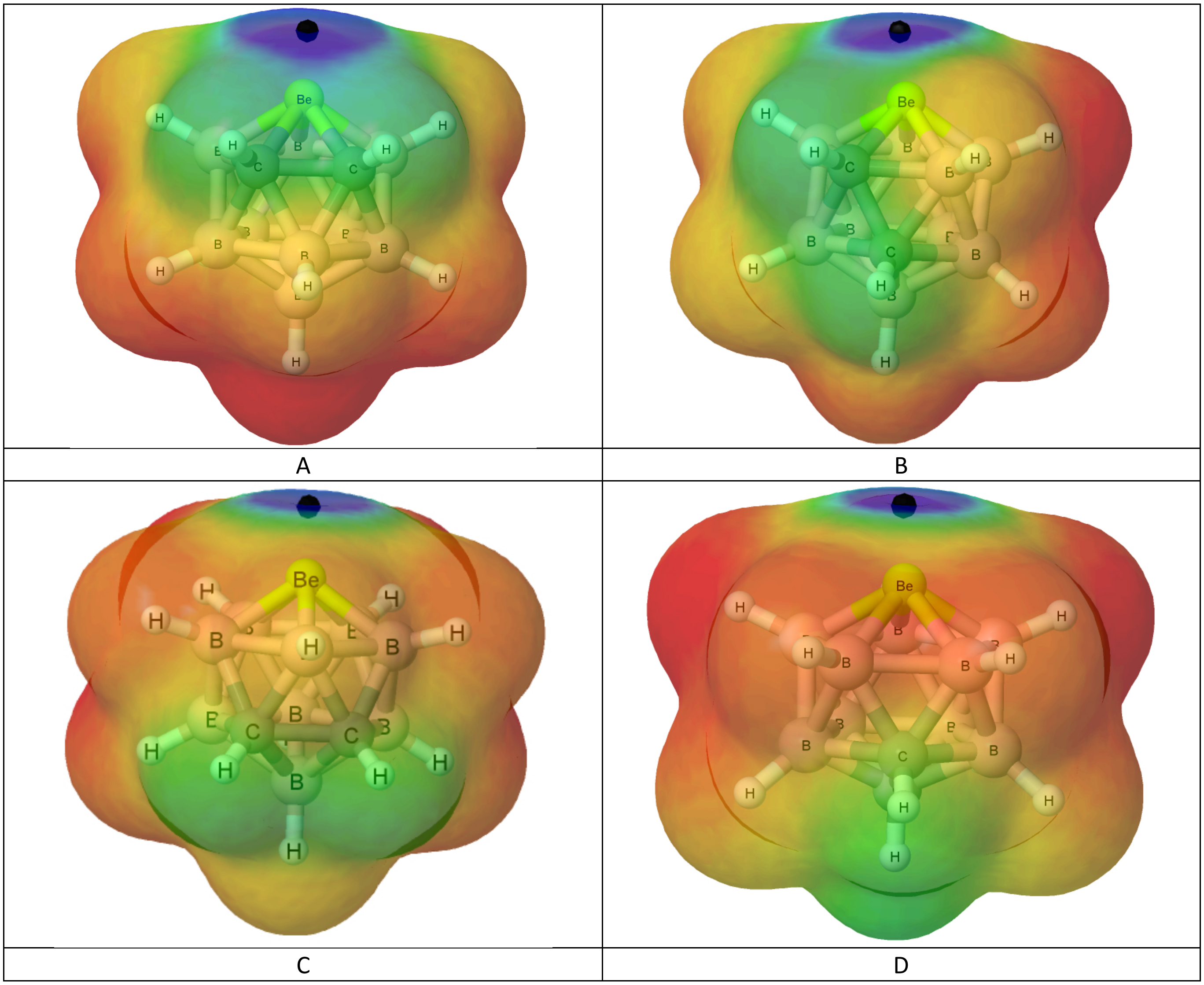

Fig. S1 Molecular electrostatic potential (MESP) on the 0.001 au electron density. Regions with MESP >0.1 au are indicated with blue color and MESP < -0.015 au with red color. The location of the $V_{S,max}$ is indicated with a black sphere.

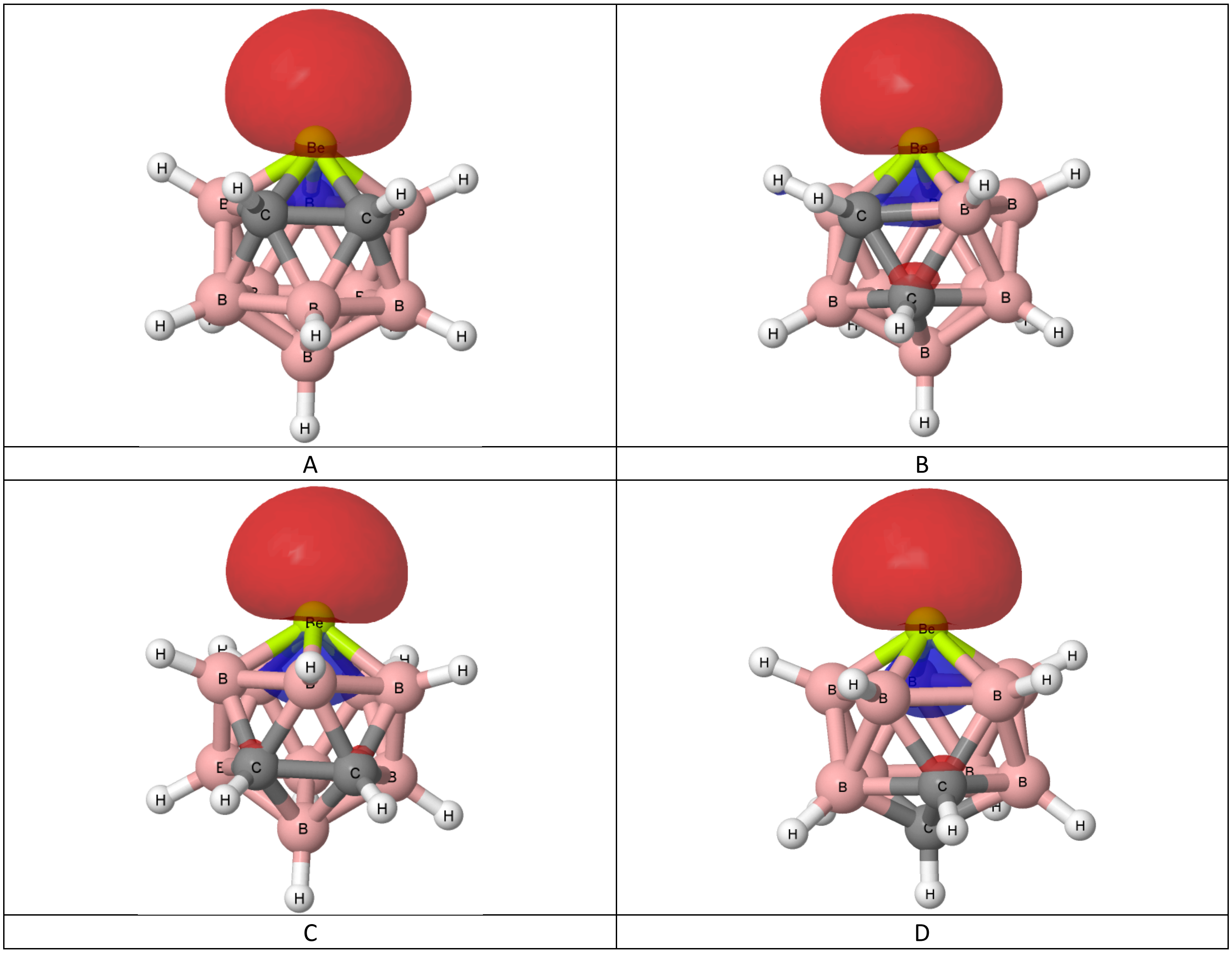

Fig. S2. LUMO of the $B_9H_{11}C_2Be$ isomers.

Table S1. Energy (a.u.) of the LUMO of the $B_9H_{11}C_2Be$ isomers.

Isomer	LUMO
**A**	-0.1048
**B**	-0.0924
**C**	-0.0786
**D**	-0.0721

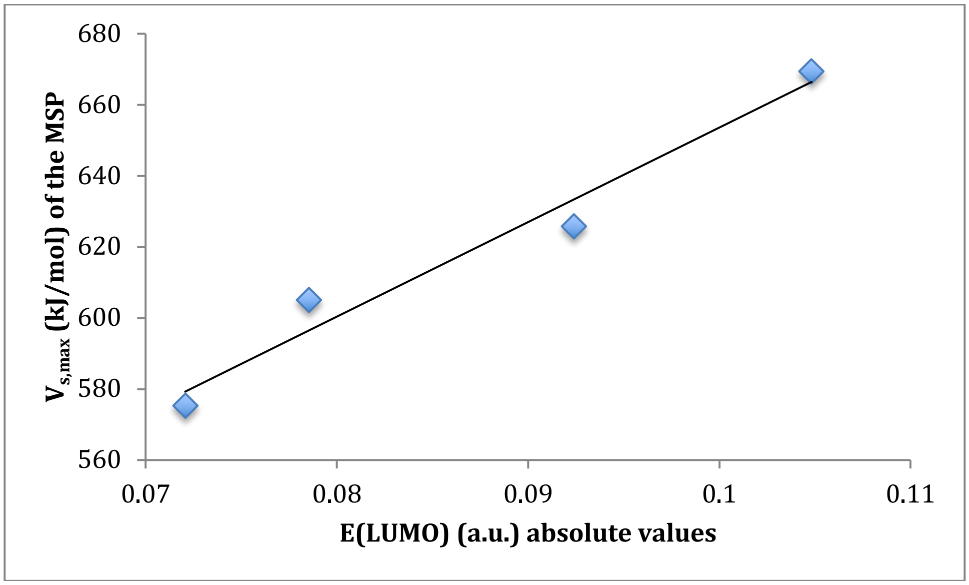

Fig. S3. Linear correlation between the $V_{s,max}$ of the MESP and the absolute value of the energy of the LUMO. The correlation obeys the equation: $V_{s,max}$ (kJ/mol) = 2664 $|E(LUMO)|$ (a.u.) +387.48 (r^2 = 0.967).

Table S2. Molecular graphs and optimized Cartesian coordinates (Å) of the complexes.

Complexes with A

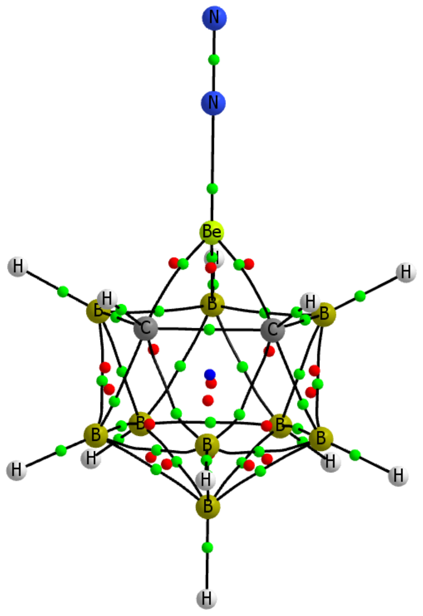	Be,-0.016714935,0.0000000135,-1.1717199127<br>B,-1.5718004778,0.0000010467,-0.1330439297<br>B,1.4550251746,-0.0000009693,1.4598640431<br>B,-0.0639932995,0.0000000401,2.3548055749<br>C,1.0781354296,0.8131600013,-0.0063612014<br>C,1.0781343476,-0.8131614344,-0.0063612036<br>B,-1.2840330717,0.8930391132,1.3913005666<br>B,-1.28403426,-0.8930374068,1.3913005641<br>B,-0.4995239922,1.444339119,-0.1050277578<br>B,-0.4995259141,-1.4443384524,-0.1050277618<br>B,0.4085939797,1.4428518442,1.4263490346<br>B,0.4085920597,-1.4428523903,1.4263490306<br>H,2.5765595406,-0.000001716,1.8252433603<br>H,0.8559921128,2.4467755144,1.8599757092<br>H,-0.0441734013,0.0000000253,3.537739294<br>H,0.855988857,-2.446776657,1.8599757025<br>H,1.9453585635,-1.300361219,-0.4348607589<br>H,-0.6484965089,2.4881900366,-0.6487793481<br>H,-2.1314299758,1.5376761272,1.9092990411<br>H,-2.1314320219,-1.5376732947,1.9092990369<br>H,-0.6484998198,-2.4881891704,-0.6487793549<br>H,-2.6422439501,0.0000017596,-0.6462522044<br>H,1.9453602938,1.3003586331,-0.4348607553<br>N,0.2061481911,-0.0000001325,-2.8490565498<br>N,0.2766538123,-0.0000001779,-3.9455190958
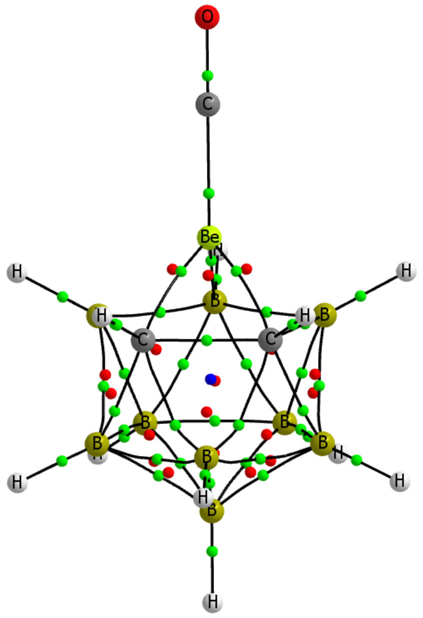	Be,0.0180848019,-0.0000000097,-1.1722777717<br>B,-1.5534118651,0.0000010319,-0.1447431478<br>B,1.4563208005,-0.0000009679,1.4810899209<br>B,-0.0741331786,0.0000000468,2.3607172896<br>C,1.0962873465,0.8134335148,0.0120271808<br>C,1.0962862668,-0.8134349687,0.0120271787<br>B,-1.2818538987,0.8934392312,1.3828087689<br>B,-1.2818550847,-0.8934375319,1.3828087665<br>B,-0.4806716354,1.4450770186,-0.1048040252<br>B,-0.4806735537,-1.4450763788,-0.1048040291<br>B,0.4105685041,1.4426034923,1.4367856535<br>B,0.4105665892,-1.4426040398,1.4367856497<br>H,2.5735190345,-0.0000017099,1.8594941837<br>H,0.8522015157,2.4469954574,1.8751466032<br>H,-0.0675827008,0.0000000408,3.5437723241<br>H,0.8521982675,-2.4469965923,1.8751465967<br>H,1.9681620208,-1.3009975873,-0.4056888761<br>H,-0.6248142494,2.4909967151,-0.6452055946<br>H,-2.1356518975,1.5379248473,1.8903341255<br>H,-2.1356539391,-1.5379220159,1.8903341214<br>H,-0.624817556,-2.4909958825,-0.6452056012<br>H,-2.6197085805,0.0000017403,-0.6660356517

	H,1.9681637478,1.3009949772,-0.4056888726<br>C,0.1465902767,-0.0000000927,-2.9027347324<br>O,0.1365197347,-0.0000000846,-4.026238963
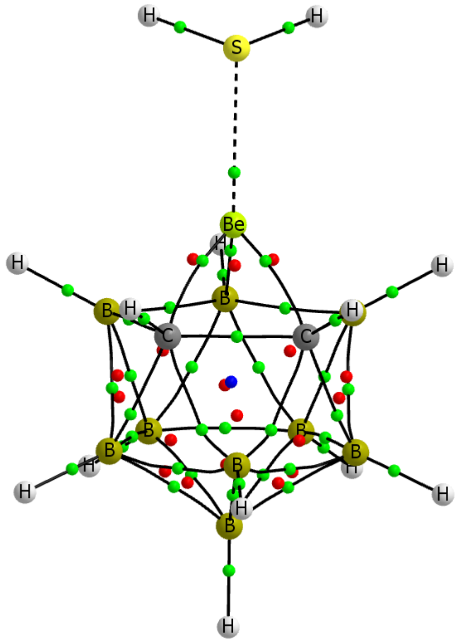	Be,1.4102580929,0.0248718012,0.<br>B,0.3127307197,1.5465293184,0.<br>B,-1.2063558237,-1.510459215,0.<br>B,-2.1374859644,-0.0175095107,0.<br>C,0.2546082182,-1.0980122739,0.8095659446<br>C,0.2546082182,-1.0980122739,-0.8095659446<br>B,-1.2043455071,1.2274851316,0.8919282912<br>B,-1.2043455071,1.2274851316,-0.8919282912<br>B,0.3129205873,0.4771608233,1.437957923<br>B,0.3129205873,0.4771608233,-1.437957923<br>B,-1.1961382195,-0.4644002973,1.4420496145<br>B,-1.1961382195,-0.4644002973,-1.4420496145<br>H,-1.545916025,-2.6406159763,0.<br>H,-1.6196055778,-0.9234390705,2.4457758184<br>H,-3.3199760431,-0.0691093514,0.<br>H,-1.6196055778,-0.9234390705,-2.4457758184<br>H,0.6991549833,-1.9567210845,-1.2976917997<br>H,0.8505452894,0.6409989362,2.4856458982<br>H,-1.7439406476,2.0610783427,1.5381775149<br>H,-1.7439406476,2.0610783427,-1.5381775149<br>H,0.8505452894,0.6409989362,-2.4856458982<br>H,0.795336464,2.6341760152,0.<br>H,0.6991549833,-1.9567210845,1.2976917997<br>S,3.5250052269,-0.2071933092,0.<br>H,3.7739257003,0.6832349774,-0.9822975985<br>H,3.7739257003,0.6832349774,0.9822975985
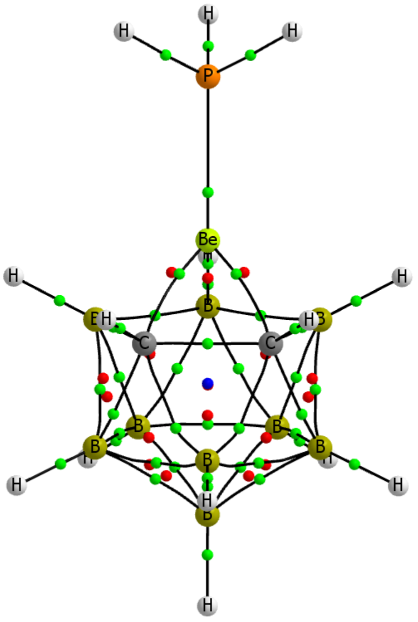	Be,0.,0.0327284593,1.072585723<br>B,0.,1.5584049475,-0.0292159878<br>B,0.,-1.4968083527,-1.5503005476<br>B,0.,-0.0022090536,-2.4825721284<br>C,-0.8103386533,-1.0851391212,-0.0910342151<br>C,0.8103386533,-1.0851391212,-0.0910342151<br>B,-0.8918830428,1.2408230396,-1.5469520956<br>B,0.8918830428,1.2408230396,-1.5469520956<br>B,-1.4386298187,0.4890003668,-0.0300063926<br>B,1.4386298187,0.4890003668,-0.0300063926<br>B,-1.4416580669,-0.4502524013,-1.5415378575<br>B,1.4416580669,-0.4502524013,-1.5415378575<br>H,0.,-2.6270046448,-1.8903185758<br>H,-2.4458279753,-0.9083108891,-1.9652679742<br>H,0.,-0.052871482,-3.6651778824<br>H,2.4458279753,-0.9083108891,-1.9652679742<br>H,1.2995435511,-1.9443728934,0.3508300711<br>H,-2.4889436561,0.6524758446,0.5009804384<br>H,-1.5379816764,2.0755819559,-2.0850566472<br>H,1.5379816764,2.0755819559,-2.0850566472

	H,2.4889436561,0.6524758446,0.5009804384<br>H,0.,2.6458203117,0.4526347045<br>H,-1.2995435511,-1.9443728934,0.3508300711<br>P,0.,-0.0454950662,3.2113681325<br>H,0.,1.2236936495,3.8173202925<br>H,-1.0781709673,-0.6451088608,3.8912990316<br>H,1.0781709673,-0.6451088608,3.8912990316
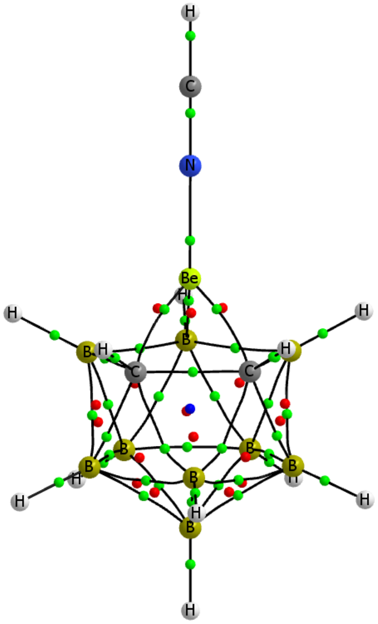	Be,-0.0538867749,0.0000000379,-1.2110449688<br>B,-1.5875228579,0.0000010551,-0.1182882849<br>B,1.4551433004,-0.0000009674,1.4311984018<br>B,-0.0471868235,0.0000000291,2.3499326714<br>C,1.0547246615,0.8095650519,-0.0312537449<br>C,1.0547235862,-0.8095664514,-0.0312537469<br>B,-1.2816359579,0.8920291024,1.4026694019<br>B,-1.2816371427,-0.8920274022,1.4026693997<br>B,-0.5172896641,1.4384712733,-0.1061455281<br>B,-0.5172915746,-1.4384705847,-0.1061455316<br>B,0.4093582134,1.4413030928,1.4131933395<br>B,0.409356299,-1.4413036386,1.413193336<br>H,2.582658111,-0.0000017166,1.7805971006<br>H,0.8637137362,2.4456958278,1.8408135322<br>H,-0.0088633744,0.0000000022,3.5331434763<br>H,0.8637104878,-2.4456969782,1.8408135262<br>H,1.9158904895,-1.297035081,-0.4713672141<br>H,-0.6758956952,2.4856149905,-0.6434684907<br>H,-2.1213527419,1.5373351604,1.934295591<br>H,-2.1213547838,-1.5373323462,1.9342955873<br>H,-0.6758989966,-2.4856140899,-0.6434684967<br>H,-2.669337471,0.0000017741,-0.6107288588<br>H,1.9158922122,1.2970325388,-0.471367211<br>N,0.1673656028,-0.000000107,-2.8566478823<br>C,0.2277360619,-0.0000001457,-3.9990022938<br>H,0.2767658132,-0.0000001769,-5.0676766038

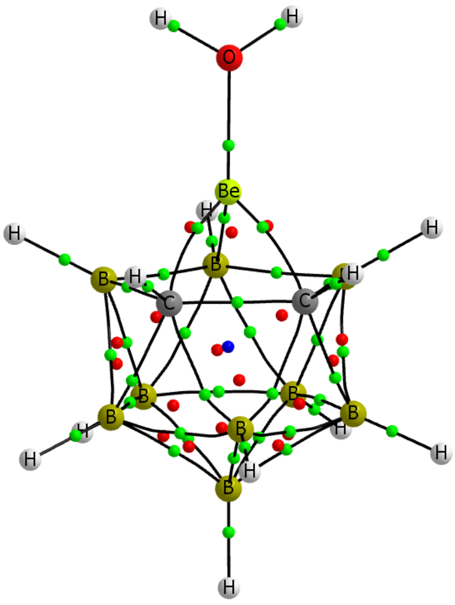	Be,1.4533686585,0.0992207367,0.<br>B,0.3123670881,1.5883563316,0.<br>B,-1.1262811062,-1.5068203584,0.<br>B,-2.0949602261,-0.0386127142,0.<br>C,0.3230671127,-1.0557195748,0.8080047031<br>C,0.3230671127,-1.0557195748,-0.8080047031<br>B,-1.194766287,1.2307162594,0.8919985214<br>B,-1.194766287,1.2307162594,-0.8919985214<br>B,0.3422309546,0.5185190345,1.4352424191<br>B,0.3422309546,0.5185190345,-1.4352424191<br>B,-1.1421171329,-0.4606074023,1.4420303704<br>B,-1.1421171329,-0.4606074023,-1.4420303704<br>H,-1.4384672927,-2.644925845,0.<br>H,-1.5552125526,-0.9297681979,2.4455169204<br>H,-3.2759472426,-0.1183989355,0.<br>H,-1.5552125526,-0.9297681979,-2.4455169204<br>H,0.7961447019,-1.9005680989,-1.2939197486<br>H,0.881286061,0.695385706,2.4815729735<br>H,-1.7570072833,2.0492665237,1.538334<br>H,-1.7570072833,2.0492665237,-1.538334<br>H,0.881286061,0.695385706,-2.4815729735<br>H,0.7717719232,2.6865985588,0.<br>H,0.7961447019,-1.9005680989,1.2939197486<br>O,3.0889738758,-0.0280567249,0.<br>H,3.504884721,0.3618255914,-0.7817796676<br>H,3.504884721,0.3618255914,0.7817796676
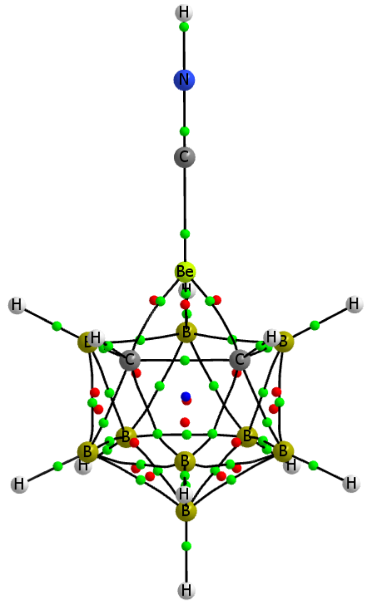	Be,-0.0289378726,0.0000000211,-1.2024073499<br>B,-1.5757418034,0.0000010436,-0.1241645918<br>B,1.454814439,-0.0000009637,1.4505270189<br>B,-0.0565615499,0.0000000355,2.3574710432<br>C,1.0678656031,0.810326579,-0.0143052826<br>C,1.0678645306,-0.8103279911,-0.0143052843<br>B,-1.2819396712,0.8922383805,1.3991882733<br>B,-1.2819408521,-0.8922366855,1.3991882714<br>B,-0.5051317472,1.4398427893,-0.1032439893<br>B,-0.5051336529,-1.4398421192,-0.1032439924<br>B,0.4092102623,1.4412893782,1.4239048977<br>B,0.4092083546,-1.4412899217,1.4239048946<br>H,2.5790097843,-0.0000017081,1.8101123166<br>H,0.8594666177,2.4457781594,1.8553193028<br>H,-0.028103063,0.0000000154,3.5409084317<br>H,0.8594633805,-2.4457792998,1.8553192974<br>H,1.9332073011,-1.2981292129,-0.4450727448<br>H,-0.6593545387,2.4886525906,-0.6376315893<br>H,-2.1264757824,1.5375880888,1.9228157882<br>H,-2.1264778175,-1.5375852772,1.9228157849<br>H,-0.6593578326,-2.4886517152,-0.6376315948<br>H,-2.6541230413,0.0000017578,-0.6231828977<br>H,1.9332090193,1.2981266564,-0.4450727419<br>C,0.1545382683,-0.0000000984,-2.9285814921

	N,0.1962170493,-0.0000001247,-4.081475474<br>H,0.2024876946,-0.0000001278,-5.0813467718
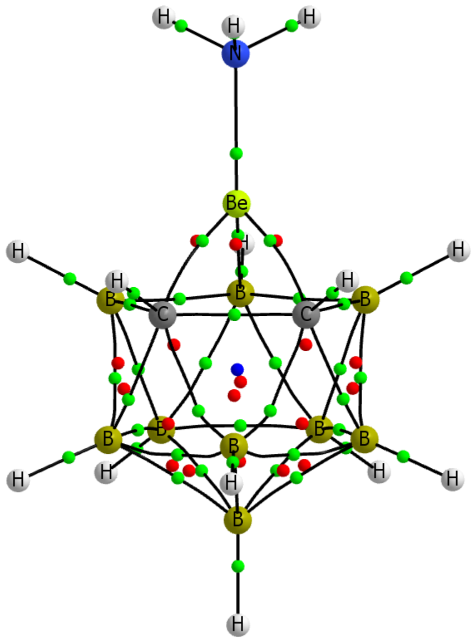	Be,1.4612473089,0.0714153898,0.<br>B,0.3192191252,1.5690719517,0.<br>B,-1.1518676912,-1.5061286239,0.<br>B,-2.1087819658,-0.0300063122,0.<br>C,0.302397135,-1.0684615478,0.8083240134<br>C,0.302397135,-1.0684615478,-0.8083240134<br>B,-1.1937766848,1.2294824998,0.8919233719<br>B,-1.1937766848,1.2294824998,-0.8919233719<br>B,0.334208908,0.5008735246,1.4346661242<br>B,0.334208908,0.5008735246,-1.4346661242<br>B,-1.160957623,-0.4604419145,1.4417326175<br>B,-1.160957623,-0.4604419145,-1.4417326175<br>H,-1.4727913709,-2.6422385918,0.<br>H,-1.5779849999,-0.9276862259,2.4448426846<br>H,-3.2906118028,-0.1005438297,0.<br>H,-1.5779849999,-0.9276862259,-2.4448426846<br>H,0.75644932,-1.92200533,-1.2979159519<br>H,0.8730407898,0.6744551597,2.4813416886<br>H,-1.7468438945,2.055471884,1.5371615039<br>H,-1.7468438945,2.055471884,-1.5371615039<br>H,0.8730407898,0.6744551597,-2.4813416886<br>H,0.7833431102,2.6655187276,0.<br>H,0.75644932,-1.92200533,1.2979159519<br>N,3.1657727053,-0.0370163733,0.<br>H,3.5541856362,-0.9781594678,0.<br>H,3.5360988874,0.4432370457,-0.8191726387<br>H,3.5360988874,0.4432370457,0.8191726387

Complexes with B

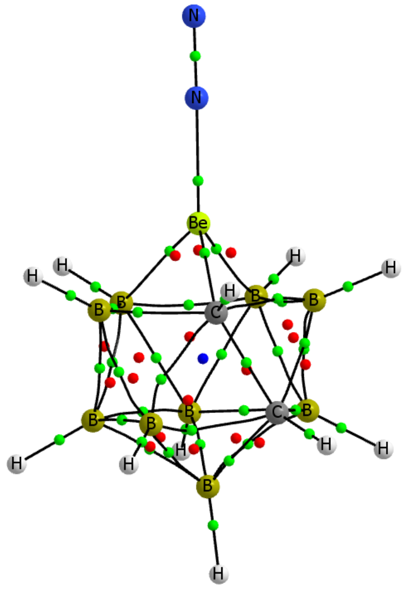	Be,-1.8554011489,0.1175753502,-0.0340302169<br>B,-0.9225865134,-1.5082003543,-0.0242202353<br>C,0.776414691,1.2515195074,-0.0134847191<br>B,1.645369375,-0.2022511017,0.0054340076<br>C,-0.6313423891,1.1320385706,0.7961414218<br>B,-0.667884114,1.2442558203,-0.9275727046<br>B,0.6032304887,-1.3391246099,0.8946810865<br>B,0.6171522217,-1.3383742017,-0.8999706999<br>B,-0.8389931328,-0.4426436817,1.4223528956<br>B,-0.8166211403,-0.4436169228,-1.493514276<br>B,0.7516638355,0.3326647798,1.4464718684<br>B,0.7738069711,0.3456077406,-1.4520667562<br>H,1.28567324,2.2015630023,0.0531356573<br>H,1.2668100375,0.8088207082,2.3946535806<br>H,2.8241088253,-0.1375644679,0.0382783826<br>H,1.3921774665,0.7723985985,-2.3639465629<br>H,-0.9795789381,2.2813945404,-1.4055383825<br>H,-1.3734362571,-0.5452357869,2.4766387871

	H,1.070811592,-2.207386782,1.5492900098<br>H,1.1067782662,-2.2202500286,-1.5200538249<br>H,-1.3024625021,-0.6982280453,-2.5464373245<br>H,-1.5017308444,-2.545170592,-0.0134414728<br>H,-0.8805972068,2.023389336,1.3574263374<br>N,-3.5164301809,0.4209857101,0.0457280025<br>N,-4.6052482817,0.5660856003,0.0739863882
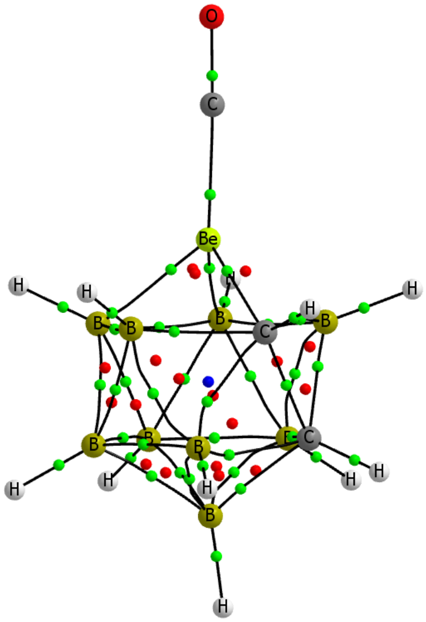	Be,-1.8559718012,0.1316116725,-0.0295101164<br>B,-0.9234470665,-1.5021670131,-0.0230298943<br>C,0.7864776155,1.2510032721,-0.0125511655<br>B,1.6504961506,-0.2074578261,0.0037457668<br>C,-0.6198329515,1.1380551303,0.7989599544<br>B,-0.659800291,1.2513455997,-0.9249139989<br>B,0.6034374754,-1.3385956792,0.8947484565<br>B,0.6163592953,-1.3379488829,-0.89997367<br>B,-0.8345401586,-0.435713158,1.4256183189<br>B,-0.8151960042,-0.4384554571,-1.4918274826<br>B,0.7602832139,0.3335544863,1.4459485516<br>B,0.7775234676,0.3464422361,-1.4515243608<br>H,1.2992637805,2.1993106874,0.0524365958<br>H,1.2781195106,0.8064917465,2.3942491482<br>H,2.8295167535,-0.1475916088,0.0352745771<br>H,1.3950370064,0.7710840915,-2.3649556095<br>H,-0.9621652748,2.290206164,-1.4041418538<br>H,-1.360420563,-0.5389530923,2.4836122893<br>H,1.0664268029,-2.2092725538,1.5493525792<br>H,1.101273757,-2.2219535537,-1.5206873989<br>H,-1.3005711801,-0.6907520667,-2.5451696914<br>H,-1.5042120091,-2.5378543546,-0.0102668579<br>H,-0.8637367704,2.0306527688,1.360192018<br>C,-3.5625154853,0.4035822224,0.0371307279<br>O,-4.6801209125,0.5236278586,0.0572243663

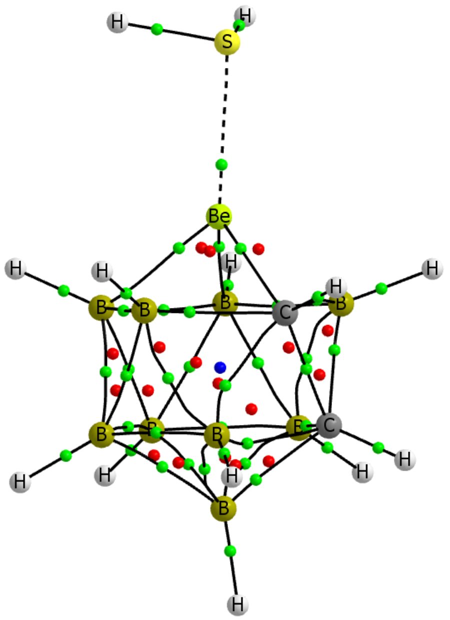	Be,-1.7965810843,0.1872003917,0.0795545621<br>B,-0.8905383443,-1.4585807079,0.0526843324<br>C,0.8863481963,1.2441737729,-0.0121965992<br>B,1.7131231318,-0.2315135455,-0.0267795869<br>C,-0.4943950232,1.1649023041,0.853492099<br>B,-0.5998312824,1.2763835238,-0.8579973977<br>B,0.6762291751,-1.3390717251,0.9050924131<br>B,0.61556628,-1.3387428586,-0.8876163425<br>B,-0.7191071438,-0.3982705859,1.4870362619<br>B,-0.8160074963,-0.4010538009,-1.4159811583<br>B,0.8942795796,0.3266307903,1.4478414858<br>B,0.7960541624,0.338954048,-1.4471003106<br>H,1.4250173895,2.1790933762,0.0319202896<br>H,1.4624670684,0.7880091427,2.3734206155<br>H,2.8940143898,-0.1990194995,-0.0423942308<br>H,1.3872898901,0.7488207976,-2.3851220416<br>H,-0.892696677,2.3240629837,-1.3291850712<br>H,-1.2035350085,-0.4883207429,2.5685954433<br>H,1.146512004,-2.2197218662,1.5421526952<br>H,1.0541406864,-2.2343771177,-1.527264748<br>H,-1.3496234431,-0.6438366425,-2.4516433811<br>H,-1.4957020216,-2.4834627947,0.0880756825<br>H,-0.6894388339,2.0630562603,1.425655366<br>S,-3.8679268938,0.6222559811,0.2679762223<br>H,-4.213362135,0.423259008,-1.0204941719<br>H,-4.2630828381,-0.6100286802,0.647535323
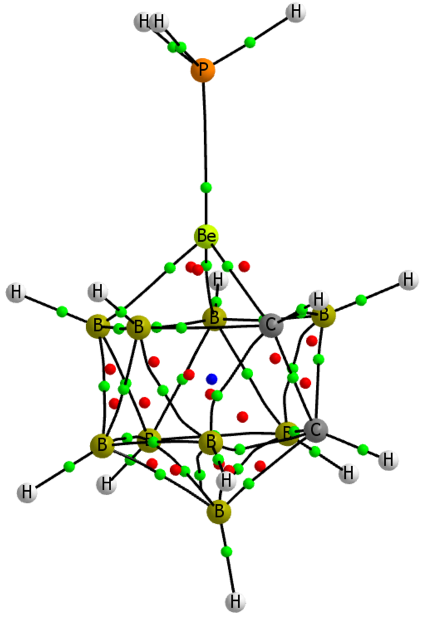	Be,-1.7500614218,0.1631282895,-0.0083576224<br>B,-0.8279362508,-1.4802614547,-0.0287326385<br>C,0.9314550094,1.234518414,0.0025061911<br>B,1.769864587,-0.235902782,-0.0048725423<br>C,-0.4770585006,1.1342752353,0.8210224991<br>B,-0.5247719194,1.269611096,-0.8932570359<br>B,0.7097382117,-1.361630538,0.8771766949<br>B,0.7083116304,-1.3367876335,-0.9163062362<br>B,-0.7105276104,-0.4400131714,1.4261192706<br>B,-0.7117064462,-0.401516656,-1.4804414256<br>B,0.8975434023,0.2971583071,1.4490542537<br>B,0.8961304416,0.3497191694,-1.4460804866<br>H,1.4612436206,2.1725456598,0.0781115646<br>H,1.4311697251,0.7502883262,2.3991546422<br>H,2.9504187565,-0.1949569968,0.0197988873<br>H,1.5153653287,0.7766020468,-2.3582050769<br>H,-0.8069705991,2.3224149077,-1.358782209<br>H,-1.2275574145,-0.5492628166,2.4900364803<br>H,1.1646066338,-2.2485344406,1.5168668925<br>H,1.1735319007,-2.2208652788,-1.5532563454<br>H,-1.2029936284,-0.6358154897,-2.5378268459<br>H,-1.4212342041,-2.5116873631,-0.0294303075<br>H,-0.6925568256,2.0238201414,1.3990991533<br>P,-3.8639139314,0.4802581845,0.0449068569

	H,-4.416505518,1.6721414743,-0.4649115053<br>H,-4.6083016218,-0.461266877,-0.6897105441<br>H,-4.5420458412,0.4136222275,1.277958616
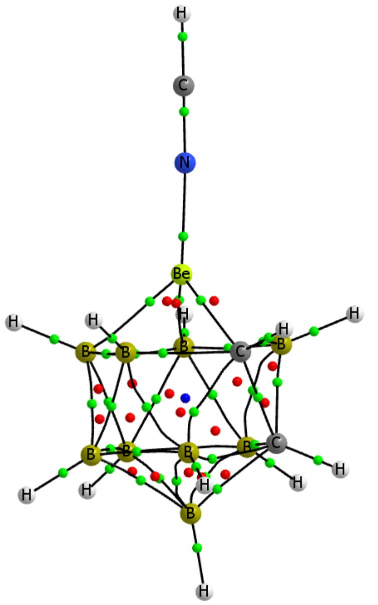	Be,-1.881261561,0.1136199015,-0.0350776055<br>B,-0.9131965752,-1.5061957984,-0.0236226006<br>C,0.7829615055,1.2494781834,-0.0141921755<br>B,1.655017432,-0.2006622013,0.0060229148<br>C,-0.6300800231,1.1259244646,0.792155102<br>B,-0.6654027876,1.2369171163,-0.921584935<br>B,0.6134676336,-1.338745635,0.8938082816<br>B,0.6265651964,-1.3382009833,-0.8995450529<br>B,-0.8320215259,-0.4433539808,1.4162595381<br>B,-0.8089840706,-0.4444705075,-1.4889493374<br>B,0.7575024429,0.3311012101,1.4446106499<br>B,0.7809108354,0.3444934311,-1.4504358735<br>H,1.2899648457,2.2006195125,0.0527640569<br>H,1.2720925123,0.8092305607,2.393160036<br>H,2.8342280032,-0.1326791525,0.0395141702<br>H,1.3985265545,0.7730721602,-2.3630654199<br>H,-0.9724801869,2.2753379219,-1.4030073756<br>H,-1.3613849689,-0.5488154941,2.4742048201<br>H,1.0834249906,-2.2061819651,1.5494387645<br>H,1.1187040597,-2.2196412046,-1.520038329<br>H,-1.2887549902,-0.7020399072,-2.5456199852<br>H,-1.4851864037,-2.5489487248,-0.0137720076<br>H,-0.8766057483,2.017175975,1.3551516289<br>N,-3.5141245104,0.4103922805,0.0427381371<br>C,-4.6499655694,0.5456886388,0.0650414595<br>H,-5.7122327291,0.6671368878,0.0839823883
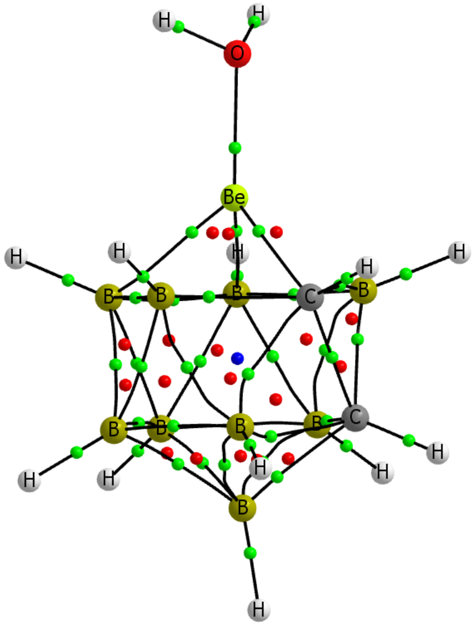	Be,-1.8506127907,0.1416795507,0.0355812598<br>B,-0.9088283216,-1.4850196745,0.0224380186<br>C,0.8214419406,1.2475194962,-0.0088457347<br>B,1.673061179,-0.2128622167,-0.0122532702<br>C,-0.5726200816,1.1424128461,0.831806118<br>B,-0.6516037921,1.2548024717,-0.8761399163<br>B,0.6412779388,-1.340991995,0.9004110796<br>B,0.6089302211,-1.3373807524,-0.8935375225<br>B,-0.7797916201,-0.4249622124,1.4567319436<br>B,-0.8298241824,-0.4219339605,-1.4427799275<br>B,0.8200436535,0.3263424743,1.4497256588<br>B,0.769878968,0.3442734504,-1.4464923693<br>H,1.3438833467,2.1909777255,0.046200315<br>H,1.3655545778,0.795370628,2.3852229622<br>H,2.8534006656,-0.1607915793,-0.0098210736<br>H,1.3707019582,0.7651666073,-2.3736440577<br>H,-0.960340312,2.2970549549,-1.3501216307<br>H,-1.289648693,-0.5238161843,2.527126069<br>H,1.1181000921,-2.2143427948,1.5429317174<br>H,1.0737124387,-2.2235776341,-1.5280254286

	H,-1.3422421494,-0.6730087681,-2.4880161225<br>H,-1.4980336652,-2.5203713266,0.0447381478<br>H,-0.7963594917,2.0349436327,1.4024837872<br>O,-3.4695489694,0.4156136731,0.1510643188<br>H,-3.9231034528,0.4567475553,-0.7015579002<br>H,-3.9282157299,-0.2330441547,0.70203131
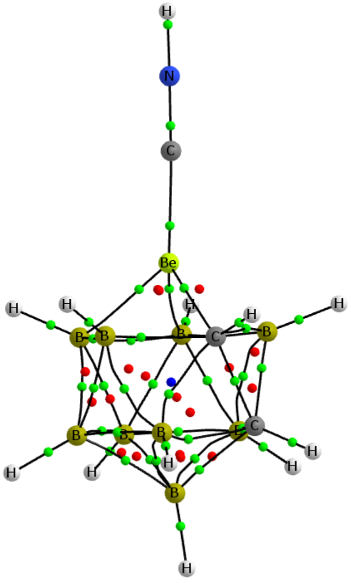	Be,-1.8724891229,0.1233009431,-0.0322476498<br>B,-0.9115145388,-1.5032521818,-0.0234060568<br>C,0.7921574321,1.2485368897,-0.0136502386<br>B,1.6604274791,-0.205096851,0.0047915255<br>C,-0.6192726347,1.1301376528,0.7943690546<br>B,-0.6570038153,1.2420994174,-0.9213885147<br>B,0.6156215072,-1.3392529254,0.8936485676<br>B,0.6286448365,-1.3387092635,-0.8996756949<br>B,-0.8270239668,-0.4395427423,1.4190881033<br>B,-0.8054034504,-0.4418485918,-1.4888502571<br>B,0.7655726479,0.3311907494,1.4442676749<br>B,0.7860290446,0.3443816798,-1.4504552836<br>H,1.3019490357,2.198308443,0.0521623912<br>H,1.2820641025,0.8068592754,2.3928767963<br>H,2.8398283944,-0.140414576,0.0376462062<br>H,1.4036317989,0.771235049,-2.3637560027<br>H,-0.9567793924,2.2817859427,-1.403438609<br>H,-1.3496200362,-0.5454899054,2.479720244<br>H,1.0824866652,-2.2081853207,1.5492715932<br>H,1.1177820354,-2.2214506492,-1.5204661218<br>H,-1.2846574018,-0.6976445258,-2.5456630287<br>H,-1.4848634358,-2.544701071,-0.0121408663<br>H,-0.8618958214,2.0223912253,1.3569820181<br>C,-3.5788997828,0.4108304914,0.0400997887<br>N,-4.7252718724,0.5452182465,0.0640416749<br>H,-5.7198153466,0.6395652883,0.0761139358

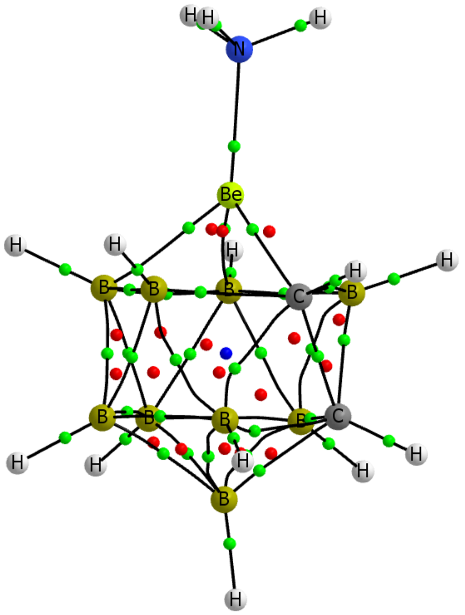

Be,-1.8601590531,0.1631616186,-0.0202067179
B,-0.9137116157,-1.4698835612,-0.0319776687
C,0.8370037168,1.2462437082,0.0023377801
B,1.6797863965,-0.2198443595,0.0011009138
C,-0.578032023,1.1402215897,0.8132270067
B,-0.6166525609,1.2722652347,-0.8940670303
B,0.6205631325,-1.3498511841,0.8788474783
B,0.6243216938,-1.3260221912,-0.9147255327
B,-0.8049117419,-0.4303679646,1.4158729452
B,-0.7951294263,-0.3930828272,-1.4821510942
B,0.7996113394,0.3080700071,1.4506420228
B,0.8109711071,0.3595859186,-1.4447373956
H,1.3641439759,2.185470724,0.0806003197
H,1.3285570288,0.765297315,2.4017924739
H,2.860307627,-0.1742648511,0.0301694161
H,1.4336041496,0.7879752923,-2.3542072538
H,-0.9059010684,2.3242417448,-1.3617912775
H,-1.3353468076,-0.5380406675,2.4757859807
H,1.0774609726,-2.2344904666,1.520821957
H,1.095884573,-2.2090125684,-1.5491256349
H,-1.2847550187,-0.6299439531,-2.5413939632
H,-1.5072581088,-2.503063624,-0.0353379864
H,-0.7944801165,2.029583541,1.3925425054
N,-3.5481227716,0.4416681132,0.0467008777
H,-3.8339762138,1.3408001687,-0.3356165711
H,-4.0104600861,-0.2786167951,-0.5063251902
H,-3.918081586,0.373502019,0.9928628199

Complexes with C

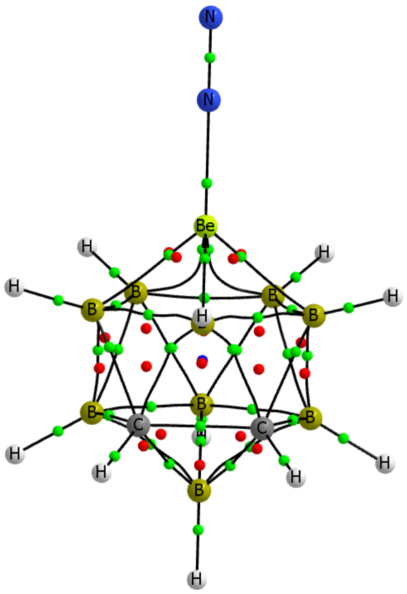

Be,1.9339497674,0.0872926669,0.
B,0.9102235921,-1.2083691461,0.9047163981
C,-0.6316539859,1.0581203817,-0.8175770987
B,-1.5881003439,-0.1126910237,0.
B,0.826485054,0.5078941236,-1.4610140738
B,0.7887489758,1.5679181386,0.
B,-0.5902573963,-1.5679851552,0.
B,-0.6306327028,-0.5211309999,1.4426436201
B,0.9102235921,-1.2083691461,-0.9047163981
B,0.826485054,0.5078941236,1.4610140738
B,-0.6306327028,-0.5211309999,-1.4426436201
C,-0.6316539859,1.0581203817,0.8175770987
H,-1.2005432427,1.8484727593,-1.2847265858
H,-1.2263783425,-0.7131472775,-2.4447576286
H,-2.7563952454,0.0445741687,0.
H,-1.2005432427,1.8484727593,1.2847265858
H,0.9842651955,2.7344415451,0.
H,1.4175985212,-2.0534761873,-1.5676344859
H,-1.1144665695,-2.6291123841,0.
H,-1.2263783425,-0.7131472775,2.4447576286

	H,1.1471103357,0.9785292091,2.5000472311<br>H,1.4175985212,-2.0534761873,1.5676344859<br>H,1.1471103357,0.9785292091,-2.5000472311<br>N,3.6185375172,0.1906396321,0.<br>N,4.7168343622,0.230069503,0.
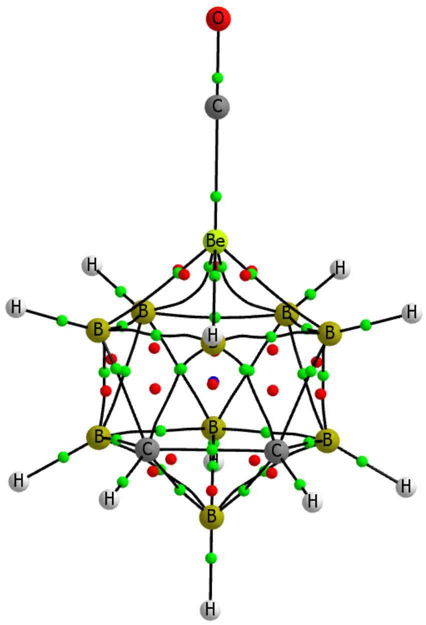	Be,1.9352662114,0.0943661004,0.<br>B,0.908209565,-1.2066511802,0.9054539419<br>C,-0.6371692314,1.0585634457,-0.8171607657<br>B,-1.5922403129,-0.1150046596,0.<br>B,0.82158067,0.5100077056,-1.462048967<br>B,0.7818860896,1.57126277,0.<br>B,-0.5918460596,-1.5678453037,0.<br>B,-0.6339842447,-0.5206463837,1.4430122685<br>B,0.908209565,-1.2066511802,-0.9054539419<br>B,0.82158067,0.5100077056,1.462048967<br>B,-0.6339842447,-0.5206463837,-1.4430122685<br>C,-0.6371692314,1.0585634457,0.8171607657<br>H,-1.2073265392,1.8479525258,-1.2844862192<br>H,-1.2289343687,-0.7136465746,-2.4453438717<br>H,-2.7608308854,0.0403169934,0.<br>H,-1.2073265392,1.8479525258,1.2844862192<br>H,0.9742424814,2.738007473,0.<br>H,1.4129743652,-2.0525040598,-1.5686062675<br>H,-1.1134702866,-2.6301857114,0.<br>H,-1.2289343687,-0.7136465746,2.4453438717<br>H,1.1392143771,0.9817290398,2.5011130957<br>H,1.4129743652,-2.0525040598,1.5686062675<br>H,1.1392143771,0.9817290398,-2.5011130957<br>C,3.6552105873,0.1846697172,0.<br>O,4.7800772257,0.2137317874,0.
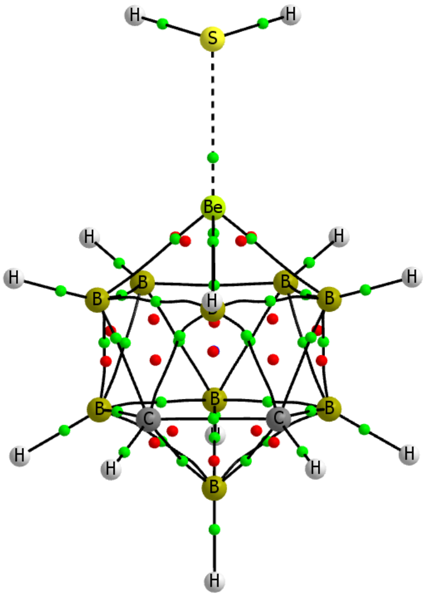	Be,-1.1119627003,-0.0334178092,0.<br>B,-0.0403040956,1.2330370992,-0.9001527537<br>C,1.4491622859,-1.0635477925,0.8169894052<br>B,2.4325407054,0.0827641105,0.<br>B,-0.0000515755,-0.4814389369,1.4553693723<br>B,0.0133441861,-1.5385677837,0.<br>B,1.4705096613,1.5617011975,0.<br>B,1.4833722929,0.5147325157,-1.4416351505<br>B,-0.0403040956,1.2330370992,0.9001527537<br>B,-0.0000515755,-0.4814389369,-1.4553693723<br>B,1.4833722929,0.5147325157,1.4416351505<br>C,1.4491622859,-1.0635477925,-0.8169894052<br>H,1.99985336,-1.8666144615,1.2841219919<br>H,2.0835653226,0.6921861901,2.4444748255<br>H,3.5972440738,-0.102067574,0.<br>H,1.99985336,-1.8666144615,-1.2841219919<br>H,-0.199329803,-2.7034261495,0.<br>H,-0.5258493464,2.0924625752,1.5665420515<br>H,2.020914837,2.6102512454,0.

	H,2.0835653226,0.6921861901,-2.4444748255<br>H,-0.3213019955,-0.9419042261,-2.500864227<br>H,-0.5258493464,2.0924625752,-1.5665420515<br>H,-0.3213019955,-0.9419042261,2.500864227<br>S,-3.2448457063,-0.1038303338,0.<br>H,-3.454731219,0.8003872841,-0.9782107467<br>H,-3.454731219,0.8003872841,0.9782107467
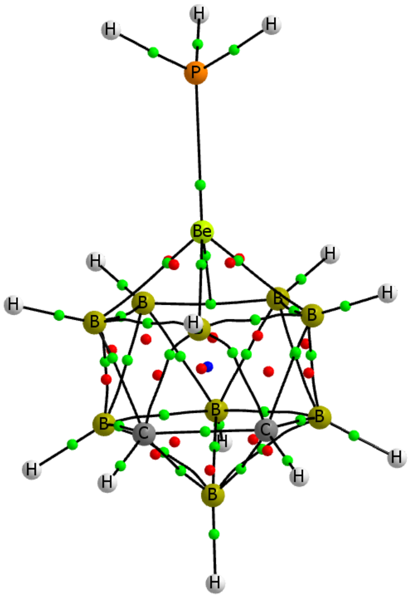	Be,0.,0.0141126368,1.0977556896<br>B,-0.9021231601,-1.2398104565,0.0054557234<br>C,0.8169410956,1.0768980806,-1.4538282452<br>B,0.,-0.0566136378,-2.4534935594<br>B,1.4566580072,0.4744053959,-0.0141451064<br>B,0.,1.5316096378,-0.0104448903<br>B,0.,-1.5470129535,-1.508922736<br>B,-1.4415201379,-0.5000828591,-1.510194106<br>B,0.9021231601,-1.2398104565,0.0054557234<br>B,-1.4566580072,0.4744053959,-0.0141451064<br>B,1.4415201379,-0.5000828591,-1.510194106<br>C,-0.8169410956,1.0768980806,-1.4538282452<br>H,1.2836800071,1.8880636454,-1.9929292091<br>H,2.4441765674,-0.6699997175,-2.1130004893<br>H,0.,0.1441314692,-3.6156582262<br>H,-1.2836800071,1.8880636454,-1.9929292091<br>H,0.,2.6944614177,0.2143811936<br>H,1.5680865286,-2.1085897495,0.4717459488<br>H,0.,-2.5885945819,-2.0724332546<br>H,-2.4441765674,-0.6699997175,-2.1130004893<br>H,-2.5008785384,0.932783216,0.3133966649<br>H,-1.5680865286,-2.1085897495,0.4717459488<br>H,2.5008785384,0.932783216,0.3133966649<br>P,0.,0.0056317157,3.2344805476<br>H,0.,-1.254869443,3.8620838277<br>H,1.0739113403,0.6057867628,3.9217276514<br>H,-1.0739113403,0.6057867628,3.9217276514

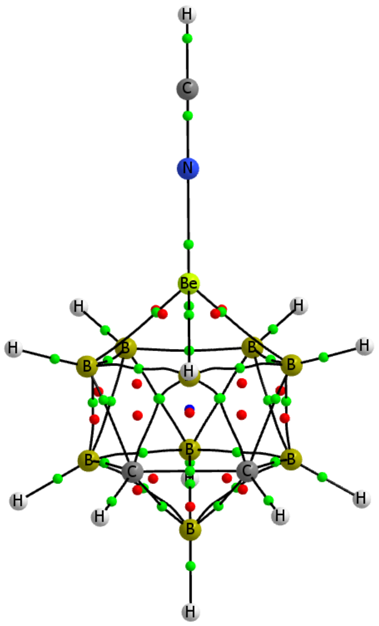

Be,1.9728539149,0.1136458461,0.
B,0.9308902919,-1.1892970253,0.9016006811
C,-0.6339446304,1.0565898803,-0.817146566
B,-1.5800095005,-0.1213553253,0.
B,0.8319791465,0.5219678078,-1.4558769464
B,0.7861430571,1.576909688,0.
B,-0.567163542,-1.5665175025,0.
B,-0.616809957,-0.5210730682,1.4411196017
B,0.9308902919,-1.1892970253,-0.9016006811
B,0.8319791465,0.5219678078,1.4558769464
B,-0.616809957,-0.5210730682,-1.4411196017
C,-0.6339446304,1.0565898803,0.817146566
H,-1.2101846255,1.8418457307,-1.2837061053
H,-1.211366079,-0.7183389133,-2.4439211121
H,-2.7502715038,0.0258377503,0.
H,-1.2101846255,1.8418457307,1.2837061053
H,0.9607238393,2.7482505836,0.
H,1.4415278806,-2.0327380751,-1.5669077297
H,-1.0826289757,-2.6329295665,0.
H,-1.211366079,-0.7183389133,2.4439211121
H,1.1403358324,0.9951470322,2.4991268756
H,1.4415278806,-2.0327380751,1.5669077297
H,1.1403358324,0.9951470322,-2.4991268756
N,3.632419984,0.217267231,0.
C,4.7763299905,0.2461632599,0.
H,5.8445713188,0.2690530946,0.

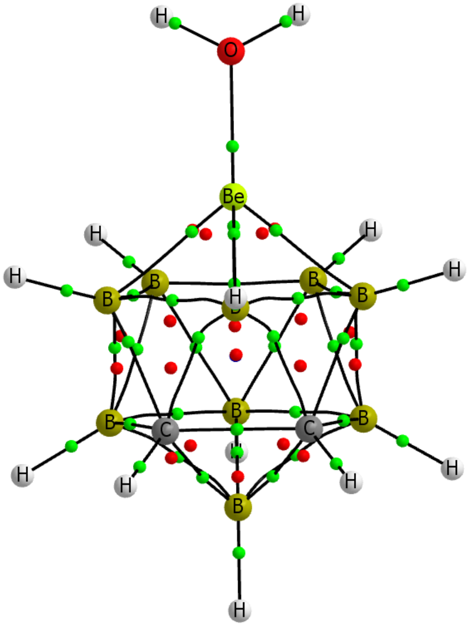

Be,-1.4877760594,-0.0737580093,0.
B,-0.434233468,1.2131509821,-0.8988505648
C,1.1021469331,-1.0486810492,0.8170312187
B,2.0611338565,0.117698645,0.
B,-0.3594465289,-0.4985646829,1.4528773348
B,-0.3233516048,-1.5547908557,0.
B,1.0687860192,1.5762862,0.
B,1.1030412044,0.5298483529,-1.4415963269
B,-0.434233468,1.2131509821,0.8988505648
B,-0.3594465289,-0.4985646829,-1.4528773348
B,1.1030412044,0.5298483529,1.4415963269
C,1.1021469331,-1.0486810492,-0.8170312187
H,1.6700565366,-1.8399187934,1.2835927107
H,1.7007151888,0.7199552165,2.4438033939
H,3.2294854096,-0.042771885,0.
H,1.6700565366,-1.8399187934,-1.2835927107
H,-0.510171129,-2.7240042564,0.
H,-0.9413506497,2.0625406748,1.5642820936
H,1.5971731867,2.6362518938,0.
H,1.7007151888,0.7199552165,-2.4438033939
H,-0.6734464053,-0.9643207603,-2.4988263203
H,-0.9413506497,2.0625406748,-1.5642820936
H,-0.6734464053,-0.9643207603,2.4988263203
O,-3.1470247135,-0.0878237603,0.

	H,-3.5225466538,0.3435278043,-0.7791426478<br>H,-3.5225466538,0.3435278043,0.7791426478
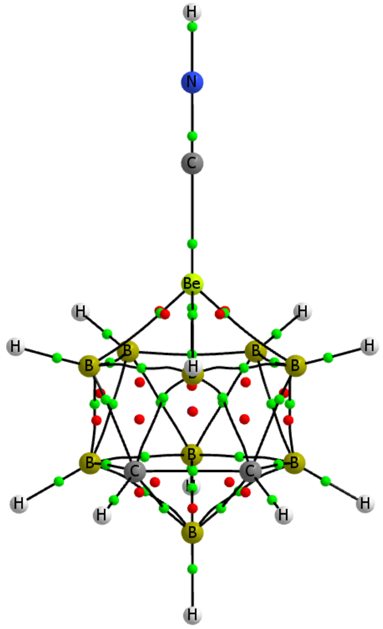	Be,1.9658035701,0.1159819589,0.<br>B,0.9264909565,-1.1904728719,0.9025475876<br>C,-0.6387019262,1.0567504626,-0.8167659989<br>B,-1.5847715661,-0.1225168333,0.<br>B,0.8269812515,0.5216977942,-1.4572436548<br>B,0.7804978558,1.5781940821,0.<br>B,-0.5715654609,-1.5667582477,0.<br>B,-0.6216139311,-0.5209688475,1.4414525441<br>B,0.9264909565,-1.1904728719,-0.9025475876<br>B,0.8269812515,0.5216977942,1.4572436548<br>B,-0.6216139311,-0.5209688475,-1.4414525441<br>C,-0.6387019262,1.0567504626,0.8167659989<br>H,-1.2151837036,1.8417512895,-1.2835417074<br>H,-1.2159101179,-0.7183378532,-2.4442400415<br>H,-2.755095534,0.0241377637,0.<br>H,-1.2151837036,1.8417512895,1.2835417074<br>H,0.95455062,2.749190547,0.<br>H,1.4339320325,-2.0349074527,-1.5680093472<br>H,-1.0863997134,-2.6333264313,0.<br>H,-1.2159101179,-0.7183378532,2.4442400415<br>H,1.1335959815,0.9954738238,2.5002047582<br>H,1.4339320325,-2.0349074527,1.5680093472<br>H,1.1335959815,0.9954738238,-2.5002047582<br>C,3.6906306636,0.2181314925,0.<br>N,4.8454361283,0.2527503236,0.<br>H,5.8437223245,0.2668498908,0.
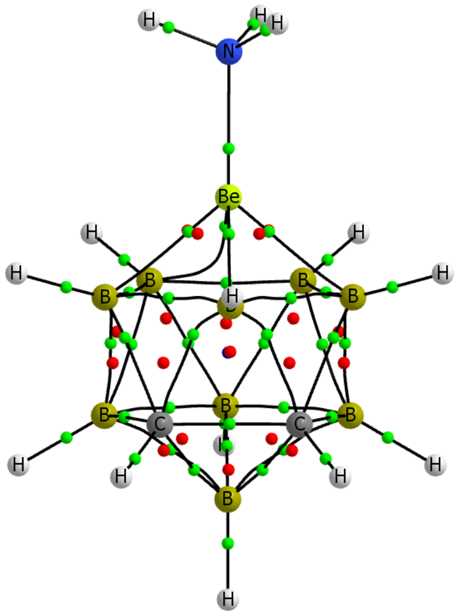	Be,-0.0055439549,1.4910019601,-0.0006894563<br>B,1.2442912039,0.3751481605,0.8908607501<br>C,-1.0822373213,-1.0772020615,-0.8109959991<br>B,0.0512678774,-2.0796459153,0.0000468264<br>B,-0.4807960131,0.3611863087,-1.4496647289<br>B,-1.524772499,0.3694265031,0.0087532327<br>B,1.5461027392,-1.1401449541,-0.0094321068<br>B,0.5087848678,-1.1373344044,1.4380149462<br>B,1.2334625305,0.3751341357,-0.9071566487<br>B,-0.4627733206,0.3622766657,1.4546936537<br>B,0.4910328739,-1.1379929761,-1.4439127493<br>C,-1.0719898072,-1.0766947418,0.8243086034<br>H,-1.8975937276,-1.6150477144,-1.2716410625<br>H,0.6522809812,-1.7422198627,-2.4475574561<br>H,-0.1543365762,-3.2411668767,0.0015712054<br>H,-1.8816573676,-1.6139956234,1.2955020587<br>H,-2.6885726453,0.596474884,0.0163917228<br>H,2.0988888832,0.8434968782,-1.5793032562<br>H,2.5853569452,-1.7086806458,-0.0159814735<br>H,0.6819076421,-1.74113465,2.4399429042<br>H,-0.9125895469,0.6907874355,2.5040685869

	H,2.1171992285,0.8473855432,1.5510457011<br>H,-0.9427576356,0.6900716658,-2.4936982357<br>N,0.0159185409,3.2094783105,0.0029149292<br>H,0.8997000566,3.5385524824,0.3868835359<br>H,-0.0642094361,3.5902875837,-0.9375361534<br>H,-0.7287735192,3.6188689094,0.5625706697

Complexes with D

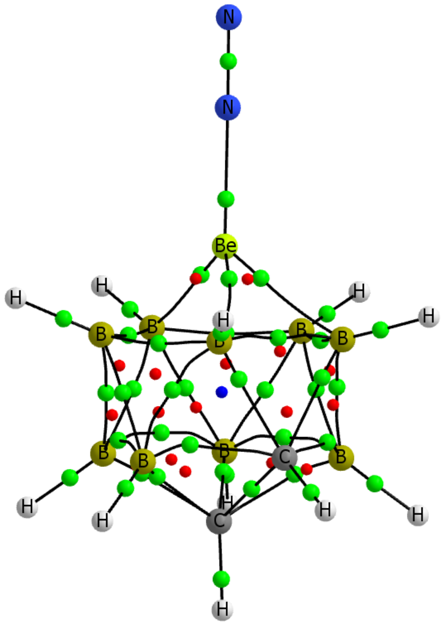	Be,-1.9019865286,-0.3992552453,0.000000005<br>B,-0.4944275528,-1.6388968449,0.0000000173<br>C,0.1990460482,1.5259528201,-0.000000016<br>C,1.3533251591,0.3776880766,-0.0000000045<br>B,-1.1514303819,1.0666378656,0.9056060798<br>B,-1.1514303826,1.0666378468,-0.9056061011<br>B,0.8914884668,-0.9864016233,0.8942360266<br>B,0.8914884661,-0.9864016419,-0.8942360068<br>B,-0.7447196008,-0.6014404402,1.474202997<br>B,-0.744719602,-0.6014404709,-1.4742029838<br>B,0.4864929706,0.6530522836,1.4591332219<br>B,0.4864929694,0.6530522532,-1.4591332359<br>H,0.562315061,2.5432863842,-0.0000000267<br>H,1.059439769,1.2174449308,2.3221368595<br>H,2.3719753393,0.7352752388,-0.0000000086<br>H,1.0594397671,1.2174448824,-2.3221368857<br>H,-1.6883995678,1.9298703669,-1.5141350009<br>H,-1.1001116289,-1.0129697152,2.5295955116<br>H,1.7407224195,-1.5675984109,1.4753074323<br>H,1.7407224183,-1.5675984416,-1.475307401<br>H,-1.100111631,-1.0129697679,-2.5295954895<br>H,-0.6706972672,-2.8135327642,0.0000000296<br>H,-1.6883995666,1.9298703984,1.5141349621<br>N,-3.5468268292,-0.790914488,0.0000000097<br>N,-4.6114255675,-1.0631980552,0.000000013

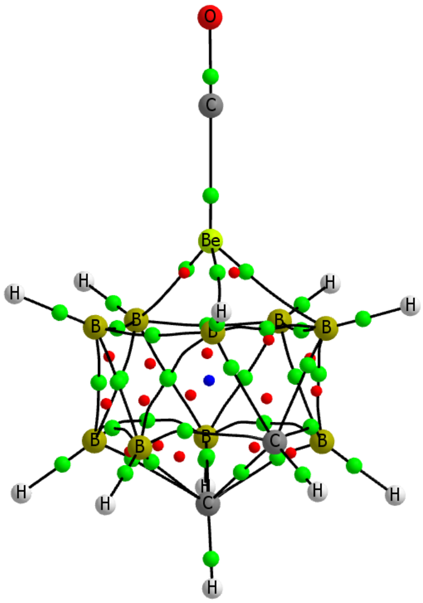	Be,-1.9059888228,-0.3928378938,0.0000000049<br>B,-0.4938800152,-1.6364222792,0.0000000172<br>C,0.2055728609,1.5279317703,-0.0000000159<br>C,1.3574535748,0.3770349318,-0.0000000045<br>B,-1.145694407,1.0719355005,0.9064283502<br>B,-1.1456944078,1.0719354817,-0.9064283715<br>B,0.8930020136,-0.9856417556,0.8941604644<br>B,0.8930020128,-0.9856417741,-0.8941604447<br>B,-0.7425994265,-0.5977372721,1.4752148211<br>B,-0.7425994277,-0.5977373027,-1.475214808<br>B,0.4909792264,0.6551535925,1.4592921761<br>B,0.4909792252,0.6551535622,-1.45929219<br>H,0.570475776,2.5447145388,-0.0000000266<br>H,1.0653019105,1.2178560259,2.3224267189<br>H,2.377062393,0.7322975577,-0.0000000086<br>H,1.0653019086,1.2178559777,-2.322426745<br>H,-1.6795246904,1.9373226111,-1.5140531098<br>H,-1.0951809372,-1.0083951173,2.5314543627<br>H,1.7406364372,-1.5688169269,1.4755001796<br>H,1.740636436,-1.5688169576,-1.4755001484<br>H,-1.0951809393,-1.0083951698,-2.5314543408<br>H,-0.6692398401,-2.8107884084,0.0000000294<br>H,-1.6795246891,1.9373226425,1.514053071<br>C,-3.5804531812,-0.809178092,0.0000000099<br>O,-4.6665802453,-1.102509803,0.0000000134
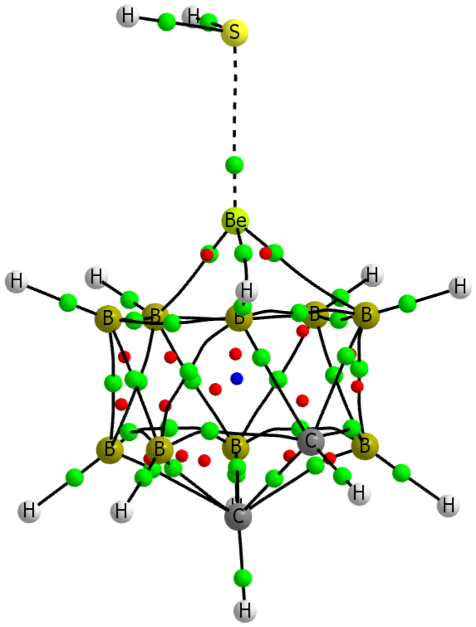	Be,0.5769844685,0.9467680536,-0.0338305343<br>B,-1.2989868883,0.8164912506,-0.0378438665<br>C,0.4309846199,-1.9157440882,0.0126210385<br>C,-1.1968998184,-1.914685613,0.0070377361<br>B,1.0642285298,-0.618720058,0.894858283<br>B,1.0710244654,-0.6504222061,-0.9083441889<br>B,-1.8330219495,-0.6066058606,0.8769684534<br>B,-1.8248759386,-0.6342543598,-0.9106346605<br>B,-0.4010760719,0.2842616365,1.4419741823<br>B,-0.3867528269,0.2356212719,-1.4950995053<br>B,-0.3920737351,-1.4718135155,1.4612475555<br>B,-0.380870599,-1.5217186337,-1.45452807<br>H,0.8882685203,-2.8942468112,0.0320694708<br>H,-0.4034673723,-2.2620245853,2.337864445<br>H,-1.6674214107,-2.8861436482,0.02136278<br>H,-0.3882451059,-2.3405302974,-2.304456708<br>H,2.0594759244,-0.9042318311,-1.5129833783<br>H,-0.4451755098,0.841543723,2.4926013503<br>H,-2.8479283166,-0.7836067445,1.4572828344<br>H,-2.8347177454,-0.8295582376,-1.4938519079<br>H,-0.428125038,0.7543929362,-2.5646395805<br>H,-2.0064063525,1.7742241768,-0.0578532592<br>H,2.0467336387,-0.8534192082,1.5175056127<br>S,1.6984819784,2.7657662522,-0.0789187191<br>H,0.7002189261,3.58010022,-0.4769773909

	H,1.5631686076,3.1149531774,1.2165680274
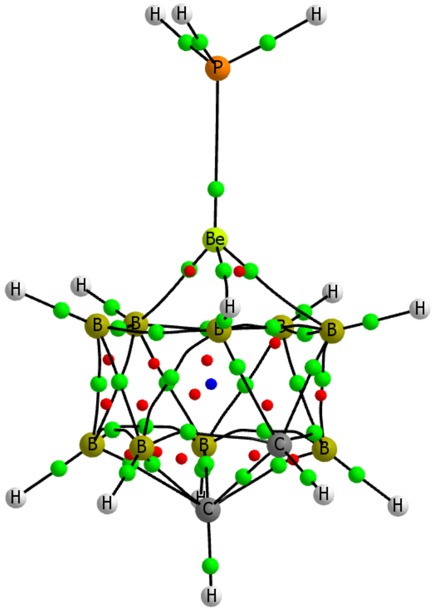	Be,-1.0936165251,0.010247456,0.<br>B,-0.0132821598,-1.5339973674,0.<br>C,1.441643617,1.3564609042,0.<br>C,2.2779703438,-0.0404426525,0.<br>B,0.0157568676,1.2426988542,0.9022251934<br>B,0.0157568676,1.2426988542,-0.9022251934<br>B,1.4921577146,-1.2476989057,0.8937703212<br>B,1.4921577146,-1.2476989057,-0.8937703212<br>B,-0.0005609332,-0.4704733519,1.4701269421<br>B,-0.0005609332,-0.4704733519,-1.4701269421<br>B,1.503102798,0.4399922881,1.4578813899<br>B,1.503102798,0.4399922881,-1.4578813899<br>H,2.045277452,2.2523458408,0.<br>H,2.1978160055,0.8460080265,2.3213899437<br>H,3.3533245907,0.0552235215,0.<br>H,2.1978160055,0.8460080265,-2.3213899437<br>H,-0.2809874756,2.2144740435,-1.5149168784<br>H,-0.4346813449,-0.7835409097,2.5319383673<br>H,2.172554858,-2.0203476432,1.4753699689<br>H,2.172554858,-2.0203476432,-1.4753699689<br>H,-0.4346813449,-0.7835409097,-2.5319383673<br>H,-0.4662970622,-2.6340887225,0.<br>H,-0.2809874756,2.2144740435,1.5149168784<br>P,-3.2337248065,0.0095606714,0.<br>H,-3.8758644836,-1.2440439724,0.<br>H,-3.9155686387,0.6164202493,1.0735653161<br>H,-3.9155686387,0.6164202493,-1.0735653161
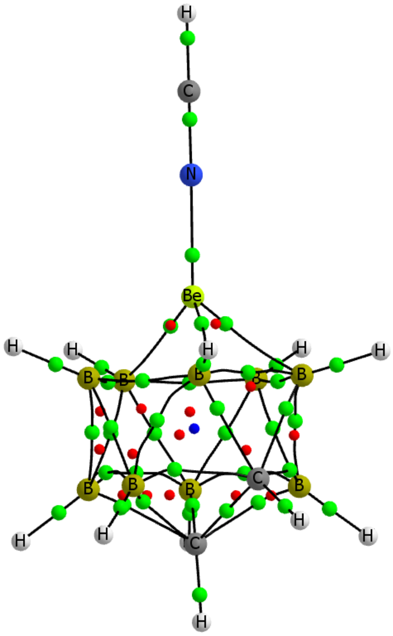	Be,-1.2419211618,-0.0012590586,0.<br>B,-0.1450826451,-1.538939668,0.<br>C,1.295381047,1.357361782,0.<br>C,2.1396740316,-0.0348625571,0.<br>B,-0.1293483242,1.2359741579,0.9017846026<br>B,-0.1293483242,1.2359741579,-0.9017846026<br>B,1.3588659088,-1.2457233227,0.8937060053<br>B,1.3588659088,-1.2457233227,-0.8937060053<br>B,-0.1367394724,-0.4759525992,1.469373332<br>B,-0.1367394724,-0.4759525992,-1.469373332<br>B,1.3617813646,0.4413222139,1.4576577666<br>B,1.3617813646,0.4413222139,-1.4576577666<br>H,1.8941627165,2.2564450584,0.<br>H,2.0549126434,0.8513150882,2.3208349124<br>H,3.2143680389,0.0666540984,0.<br>H,2.0549126434,0.8513150882,-2.3208349124<br>H,-0.4333553151,2.2060321364,-1.5133604925<br>H,-0.5750356974,-0.7906209831,2.528622423<br>H,2.0433433073,-2.0154105753,1.4749033017<br>H,2.0433433073,-2.0154105753,-1.4749033017<br>H,-0.5750356974,-0.7906209831,-2.528622423

	H,-0.594251337,-2.6402790345,0.<br>H,-0.4333553151,2.2060321364,1.5133604925<br>N,-2.9065631775,0.0130622504,0.<br>C,-4.0506596112,-0.0037968012,0.<br>H,-5.1190434645,-0.022063111,0.
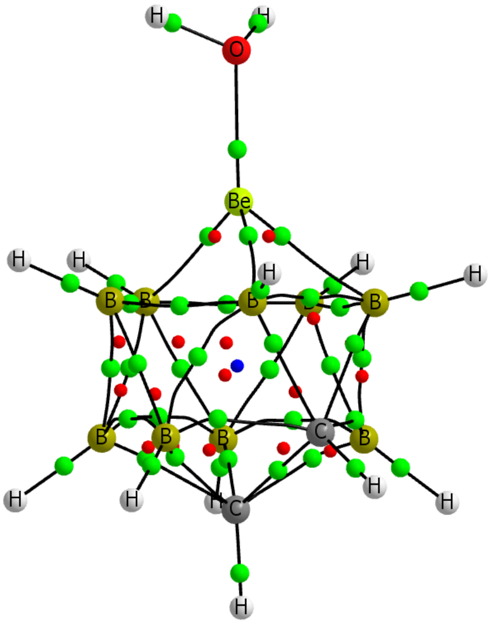	Be,-1.8893213556,-0.5825374034,0.0000000069<br>B,-0.3793897186,-1.7113310423,0.0000000178<br>C,0.1079711644,1.4838680297,-0.0000000153<br>C,1.3351496268,0.4146623021,-0.0000000049<br>B,-1.2150442145,0.9356862979,0.8993371031<br>B,-1.2150442153,0.9356862794,-0.8993371212<br>B,0.9625503862,-0.9765591192,0.8937906154<br>B,0.9625503853,-0.9765591376,-0.8937905962<br>B,-0.6996614714,-0.6985365283,1.4652254884<br>B,-0.6996614727,-0.6985365584,-1.4652254733<br>B,0.4473739366,0.6313172906,1.4578182936<br>B,0.4473739352,0.6313172606,-1.457818307<br>H,0.4061810476,2.5221259408,-0.0000000261<br>H,0.98321787,1.2312868371,2.3216535981<br>H,2.327704238,0.8387500318,-0.0000000097<br>H,0.9832178678,1.2312867893,-2.3216536243<br>H,-1.7985871518,1.7651002455,-1.5151086705<br>H,-1.023497881,-1.1341673775,2.5254765022<br>H,1.8487885877,-1.4999097861,1.4762038318<br>H,1.8487885863,-1.4999098165,-1.4762038026<br>H,-1.0234978834,-1.1341674295,-2.5254764779<br>H,-0.4757804244,-2.8991162998,0.0000000301<br>H,-1.7985871504,1.7651002767,1.5151086358<br>O,-3.4552035574,-1.1319901927,0.0000000133<br>H,-3.6715828895,-1.6591842929,-0.7803547146<br>H,-3.6715828887,-1.6591842768,0.7803547522
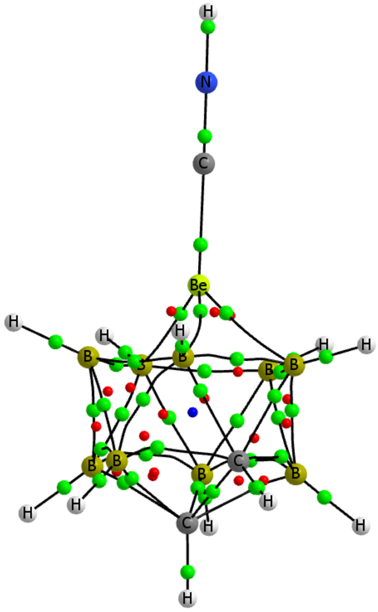	Be,-1.9465451141,-0.2529141225,-0.0010083761<br>B,-0.5899482229,-1.5689288381,0.0004308974<br>C,0.3004206669,1.5421394771,-0.0011993264<br>C,1.382873421,0.3257422091,-0.0000348499<br>B,-1.0788455159,1.1655544446,0.9014252462<br>B,-1.0781831873,1.1644344321,-0.9044112827<br>B,0.83504035,-1.0059758673,0.8943349356<br>B,0.8356771396,-1.0071059431,-0.8931244567<br>B,-0.7758161052,-0.5204169921,1.4703323169<br>B,-0.7747294349,-0.5222758709,-1.4709715887<br>B,0.531092745,0.6544454089,1.4573276616<br>B,0.5321554482,0.6526115607,-1.4584370105<br>H,0.7259869564,2.535030661,-0.0016717962<br>H,1.1381568846,1.1835034849,2.3204010148<br>H,2.4215524912,0.6202122587,0.0001389214<br>H,1.1398655398,1.180561026,-2.3217328928<br>H,-1.5521134168,2.0632609174,-1.5158644526<br>H,-1.1472370683,-0.908547539,2.5301899625

	H,1.6473619045,-1.6382170452,1.4764007263<br>H,1.6484248738,-1.6400762748,-1.4738003575<br>H,-1.1452921496,-0.9117631955,-2.5306262382<br>H,-0.8292757639,-2.7334650768,0.0010879476<br>H,-1.553244624,2.0651248793,1.5114241647<br>C,-3.6485457959,-0.5651319122,0.0011765918<br>N,-4.7804909239,-0.7968979036,0.0031046171<br>H,-5.7561698756,-1.007875263,0.0051076295
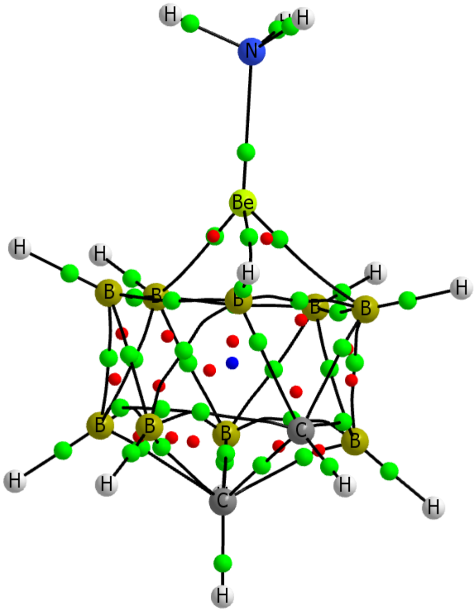	Be,-1.8970907874,-0.5869237864,0.0000000069<br>B,-0.3746179555,-1.7073896763,0.0000000179<br>C,0.1139577267,1.4860702399,-0.0000000154<br>C,1.3431731453,0.4189047112,-0.0000000049<br>B,-1.206318822,0.9329760246,0.8993816717<br>B,-1.2063188228,0.932976006,-0.8993816899<br>B,0.9701696491,-0.9732736589,0.8940358753<br>B,0.9701696483,-0.9732736774,-0.894035856<br>B,-0.690207318,-0.6957347691,1.4653975728<br>B,-0.6902073193,-0.6957347994,-1.4653975578<br>B,0.4572109663,0.6336318225,1.4582046568<br>B,0.457210965,0.6336317923,-1.4582046703<br>H,0.4107974778,2.5247265203,-0.0000000263<br>H,0.9922183257,1.2369043742,2.3206218947<br>H,2.3351112794,0.8446270877,-0.0000000098<br>H,0.9922183236,1.2369043262,-2.3206219211<br>H,-1.7895747852,1.7659452184,-1.5136982882<br>H,-1.0121265265,-1.1304835858,2.5264349908<br>H,1.8577666728,-1.4967644072,1.474701034<br>H,1.8577666715,-1.4967644378,-1.4747010046<br>H,-1.0121265287,-1.1304836381,-2.5264349665<br>H,-0.463932951,-2.8956434714,0.0000000302<br>H,-1.7895747839,1.7659452497,1.5136982533<br>N,-3.5316042295,-1.1209994631,0.0000000132<br>H,-4.1991619787,-0.3530403048,0.0000000055<br>H,-3.7248223369,-1.6926378524,-0.8198176976<br>H,-3.7248223362,-1.6926378354,0.8198177359

Complexes with SO_4Be

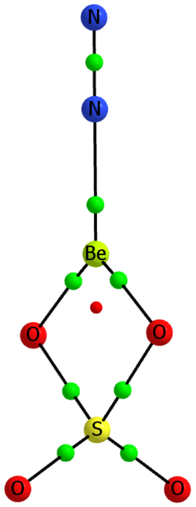	Be<br>X,1,1.<br>X,1,1.,2,90.<br>O,1,r1,2,a1,3,90.,0<br>O,1,r1,2,a1,3,-90.,0<br>S,1,r2,3,90.,2,0.,0<br>O,6,r11,1,a11,3,0.,0<br>O,6,r11,1,a11,3,180.,0<br>N,1,r20,3,90.,2,180.,0<br>N,1,r21,3,90.,2,180.,0<br><br>r1=1.49184134<br>a1=50.16290343<br>r11=1.42969953<br>a11=119.68336993<br>r2=2.10148417<br>r20=1.72944741<br>r21=2.82474555
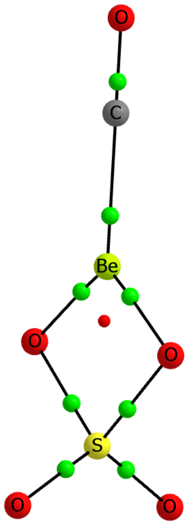	Be<br>X,1,1.<br>X,1,1.,2,90.<br>O,1,r1,2,a1,3,90.,0<br>O,1,r1,2,a1,3,-90.,0<br>S,1,r2,3,90.,2,0.,0<br>O,6,r11,1,a11,3,0.,0<br>O,6,r11,1,a11,3,180.,0<br>C,1,r20,3,90.,2,180.,0<br>O,1,r21,3,90.,2,180.,0<br><br>r1=1.49717897<br>a1=49.90596691<br>r11=1.42997602<br>a11=119.71694721<br>r2=2.10786612<br>r20=1.80188171<br>r21=2.9197351

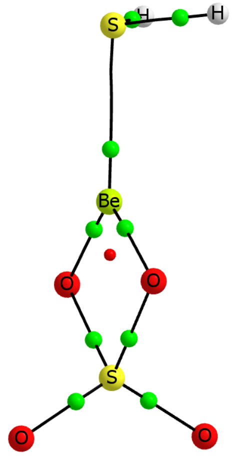	Be,-0.1963680949,0.,-1.1488441609<br>O,-0.0994294136,1.1441795513,-0.1796836494<br>O,-0.0994294136,-1.1441795513,-0.1796836494<br>S,0.0153593334,0.,0.9528122063<br>O,-1.1447962425,0.,1.7910113839<br>O,1.3230439272,0.,1.5361132929<br>S,-0.3176724137,0.,-3.2578717928<br>H,0.5794228482,0.9875198576,-3.4629891295<br>H,0.5794228482,-0.9875198576,-3.4629891295
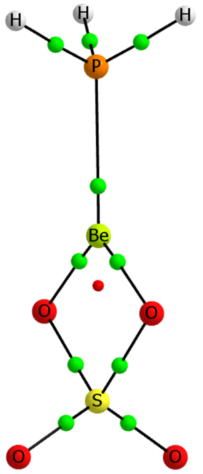	Be,0.7719706738,0.0248460201,0.0014551413<br>O,-0.2099194018,0.1758091404,-1.1309645684<br>O,-0.2081075657,-0.1478012741,1.1323494185<br>S,-1.344805731,-0.0006366674,-0.0004888376<br>O,-2.0450395696,-1.2373786899,-0.1767591221<br>O,-2.0770687689,1.2175569676,0.1742750849<br>P,2.9356601555,-0.0031617863,-0.0042550283<br>H,3.5925490435,0.7476228862,-0.9953266301<br>H,3.5942218813,0.4399224733,1.1565105361<br>H,3.5381282828,-1.26170507,-0.1846869944
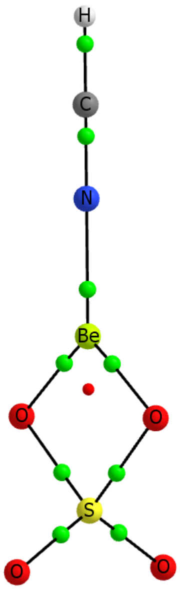	Be<br>X,1,1.<br>X,1,1.,2,90.<br>O,1,r1,2,a1,3,90.,0<br>O,1,r1,2,a1,3,-90.,0<br>S,1,r2,3,90.,2,0.,0<br>O,6,r11,1,a11,3,0.,0<br>O,6,r11,1,a11,3,180.,0<br>N,1,r20,3,90.,2,180.,0<br>C,1,r21,3,90.,2,180.,0<br>H,1,r22,3,90.,2,180.,0<br><br>r1=1.50761796<br>a1=49.26302413<br>r11=1.43250865<br>a11=120.09561047<br>r2=2.11693735<br>r20=1.66955813<br>r21=2.81134635<br>r22=3.88250424

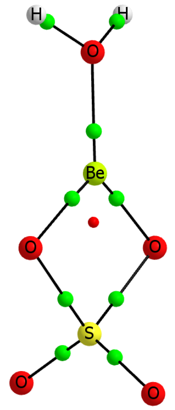	Be,-0.0367074401,0.,-1.2654795202<br>O,-0.0260328731,1.1442473587,-0.287684158<br>O,-0.0260328731,-1.1442473587,-0.287684158<br>S,-0.0027130175,0.,0.8459390767<br>O,-1.2252459373,0.,1.5912813074<br>O,1.2536522417,0.,1.5343034573<br>O,0.0591663,0.,-2.8592109182<br>H,0.3219765561,0.7937796942,-3.3417783701<br>H,0.3219765561,-0.7937796942,-3.3417783701
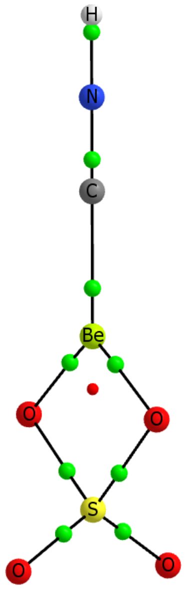	Be<br>X,1,1.<br>X,1,1.,2,90.<br>O,1,r1,2,a1,3,90.,0<br>O,1,r1,2,a1,3,-90.,0<br>S,1,r2,3,90.,2,0.,0<br>O,6,r11,1,a11,3,0.,0<br>O,6,r11,1,a11,3,180.,0<br>C,1,r20,3,90.,2,180.,0<br>N,1,r21,3,90.,2,180.,0<br>H,1,r22,3,90.,2,180.,0<br><br>r1=1.50856025<br>a1=49.23879426<br>r11=1.43226129<br>a11=120.06010634<br>r2=2.11879626<br>r20=1.77121222<br>r21=2.92090301<br>r22=3.92270494
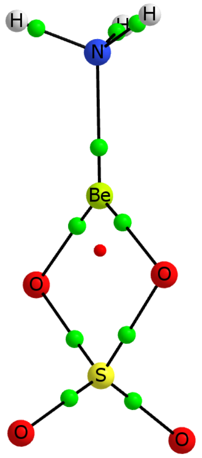	Be,0.0028421533,-1.2448211107,-0.0085991001<br>O,0.0921308299,-0.2587050614,1.1341917824<br>O,-0.0900234983,-0.2513403005,-1.1431925605<br>S,-0.0010059546,0.8761781677,0.0000524736<br>O,1.2330156366,1.598116754,-0.0966484693<br>O,-1.2377765183,1.5928184107,0.100959262<br>N,0.0075496475,-2.9361237383,0.0048290775<br>H,-0.768558967,-3.3062992556,0.5532987562<br>H,-0.0660797183,-3.326944472,-0.9338377169<br>H,0.8620103893,-3.3027723939,0.4239464953

Complexes with CO_3Be

<table>
<tr>
<td>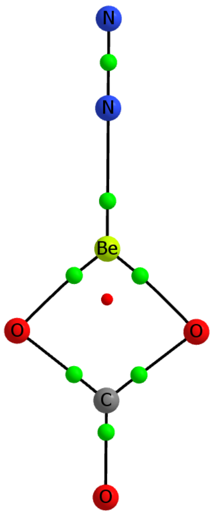
</td>
<td>Be
X,1,1.
X,1,1.,2,90.
O,1,r1,2,a1,3,90.,0
O,1,r1,2,a1,3,-90.,0
C,1,r2,3,90.,2,0.,0
O,1,r21,3,90.,2,0.,0
N,1,r10,3,90.,2,180.,0
N,1,r11,3,90.,2,180.,0

r1=1.48835342
a1=47.22160079
r2=1.85829487
r21=3.04657425
r10=1.72418875
r11=2.82032273</td>
</tr>
<tr>
<td>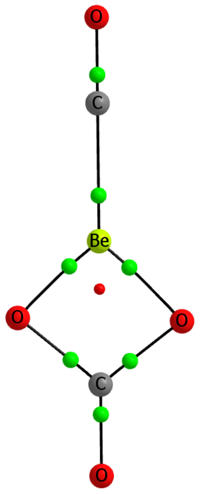
</td>
<td>Be
X,1,1.
X,1,1.,2,90.
O,1,r1,2,a1,3,90.,0
O,1,r1,2,a1,3,-90.,0
C,1,r2,3,90.,2,0.,0
O,1,r21,3,90.,2,0.,0
C,1,r10,3,90.,2,180.,0
O,1,r11,3,90.,2,180.,0

r1=1.49437121
a1=46.93238639
r2=1.86529118
r21=3.0542137
r10=1.79407564
r11=2.91337923</td>
</tr>
<tr>
<td>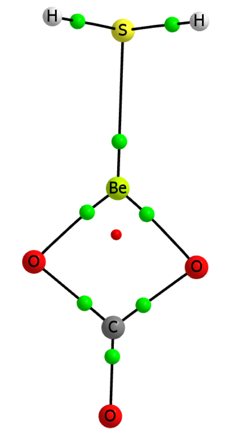
</td>
<td>Be,-0.1753354445,0.,0.6738509965
O,-0.0632650038,1.0917854588,-0.342672081
O,-0.0632650038,-1.0917854588,-0.342672081
C,0.0315601879,0.,-1.1828198926
O,0.168134488,0.,-2.3651209044
S,-0.3451001131,0.,2.784716655
H,0.5437824832,0.9870772072,3.0224716511
H,0.5437824832,-0.9870772072,3.0224716511</td>
</tr>
</table>

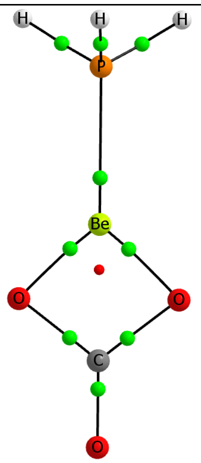	Be,-0.011335693,0.2168309779,0.0253920571<br>O,1.1173961396,-0.7412142493,-0.2156192382<br>O,-1.0116627624,-0.8808007436,0.2363567247<br>C,0.105216917,-1.6528265998,-0.0030324134<br>O,0.1791039991,-2.8413623689,-0.0220369927<br>P,-0.1585087605,2.3788424346,-0.0004734139<br>H,-1.137541563,2.9838577947,0.8085886228<br>H,-0.4602763333,2.9642900853,-1.244364531<br>H,0.9798640564,3.1225906692,0.3601891848
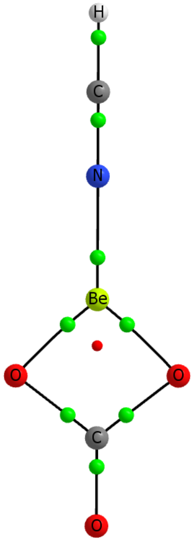	Be<br>X,1,1.<br>X,1,1.,2,90.<br>O,1,r1,2,a1,3,90.,0<br>O,1,r1,2,a1,3,-90.,0<br>C,1,r2,3,90.,2,0.,0<br>O,1,r21,3,90.,2,0.,0<br>N,1,r10,3,90.,2,180.,0<br>C,1,r11,3,90.,2,180.,0<br>H,1,r12,3,90.,2,180.,0<br><br>r1=1.50178367<br>a1=46.47869219<br>r2=1.87553959<br>r21=3.06772195<br>r10=1.67073546<br>r11=2.81318081<br>r12=3.88367789
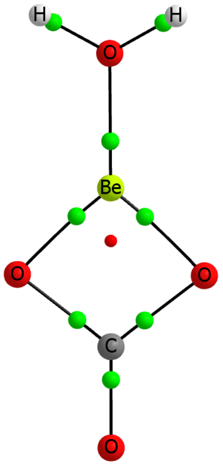	Be,-0.0299173482,0.,0.8109471756<br>O,-0.0169609194,1.0912686981,-0.2154808921<br>O,-0.0169609194,-1.0912686981,-0.2154808921<br>C,0.0009514699,0.,-1.0589386034<br>O,0.025654713,0.,-2.2501088839<br>O,0.0492623368,0.,2.4115166066<br>H,0.3139861509,0.7930390062,2.8933077959<br>H,0.3139861509,-0.7930390062,2.8933077959

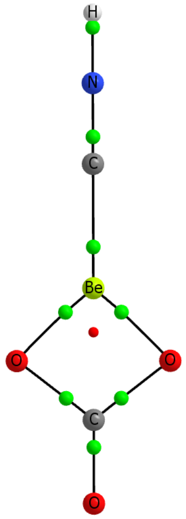	Be<br>X,1,1.<br>X,1,1.,2,90.<br>O,1,r1,2,a1,3,90.,0<br>O,1,r1,2,a1,3,-90.,0<br>C,1,r2,3,90.,2,0.,0<br>O,1,r21,3,90.,2,0.,0<br>C,1,r10,3,90.,2,180.,0<br>N,1,r11,3,90.,2,180.,0<br>H,1,r12,3,90.,2,180.,0<br><br>r1=1.50339133<br>a1=46.41851026<br>r2=1.87741437<br>r21=3.06930933<br>r10=1.76962864<br>r11=2.92059503<br>r12=3.92159201
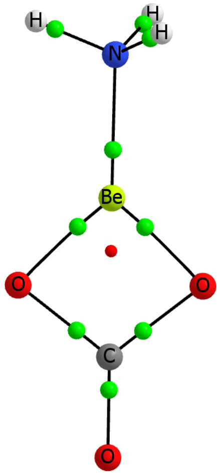	Be,-0.00007441,0.7812307151,0.0009239622<br>O,1.083408394,-0.2501166396,0.1569687397<br>O,-1.0722084264,-0.2629233971,-0.156779457<br>C,0.0101708496,-1.0989512669,-0.0005883126<br>O,0.0177023946,-2.2913813414,-0.0013997488<br>N,-0.0255684319,2.4779957409,-0.0000951477<br>H,-0.628515251,2.8408966931,0.7375580579<br>H,-0.3886888713,2.8435373831,-0.8798111982<br>H,0.9032957523,2.8737581129,0.1382231045

Complexes with Cl_2Be

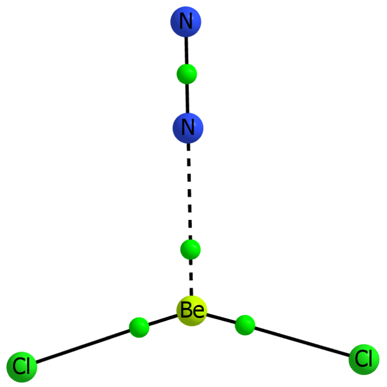	Be<br>X,1,1.<br>Cl,1,r1,2,a1<br>Cl,1,r1,2,a1,3,180.,0<br>N,1,r2,3,a1,2,0.,0<br>N,1,r3,3,a1,2,0.,0<br><br>r1=1.84266688<br>r2=1.88424314<br>r3=2.98085024<br>a1=107.10147857
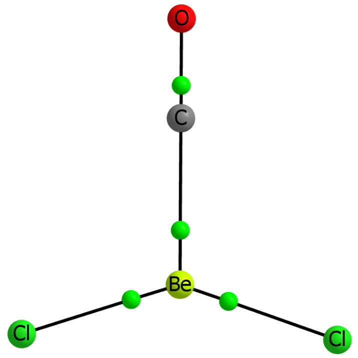	Be<br>X,1,1.<br>Cl,1,r1,2,a1<br>Cl,1,r1,2,a1,3,180.,0<br>C,1,r2,3,a1,2,0.,0<br>O,1,r3,3,a1,2,0.,0<br><br>r1=1.85398427<br>r2=1.87414442<br>r3=2.99642107<br>a1=108.51427732
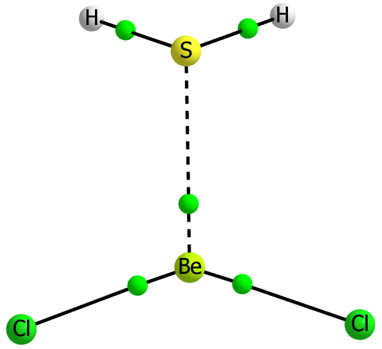	Be<br>X,1,1.<br>Cl,1,r1,2,a1<br>Cl,1,r1,2,a1,3,180.,0<br>S,1,r2,3,a1,2,0.,0<br>H,5,r4,1,a4,3,d4,0<br>H,5,r4,1,a4,4,-d4,0<br><br>r1=1.8572488<br>r2=2.24956836<br>r4=1.34671404<br>a1=109.46201323<br>a4=99.15363265<br>d4=41.99516344
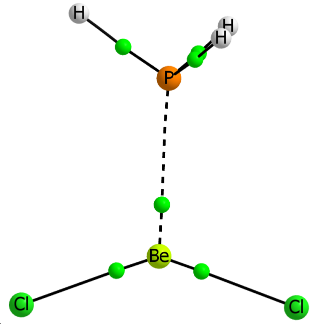	Be,0.0564350757,-0.0131945912,0.0366609623<br>Cl,1.6928658127,-0.4755215687,-0.7269386539<br>Cl,-1.714799149,0.2585381715,-0.4536131029<br>P,0.4151213495,0.300406836,2.2639378646<br>H,-0.6141525977,0.6641697281,3.156597353<br>H,1.3758817726,1.2713231648,2.6137818464<br>H,0.9560234527,-0.792879245,2.9712674345

<table>
<tr><td>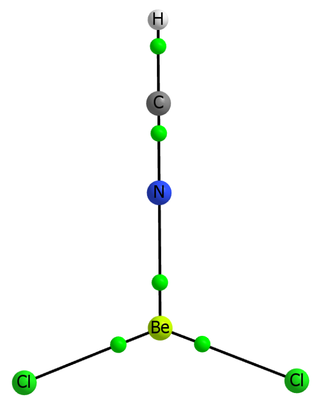
</td><td>Be
X,1,1.
Cl,1,r1,2,a1
Cl,1,r1,2,a1,3,180.,0
N,1,r2,3,a1,2,0.,0
C,1,r3,3,a1,2,0.,0
H,1,r4,3,a1,2,0.,0

r1=1.8676787
r2=1.73035406
r3=2.87350644
r4=3.94276406
a1=111.56446494</td></tr>
<tr><td>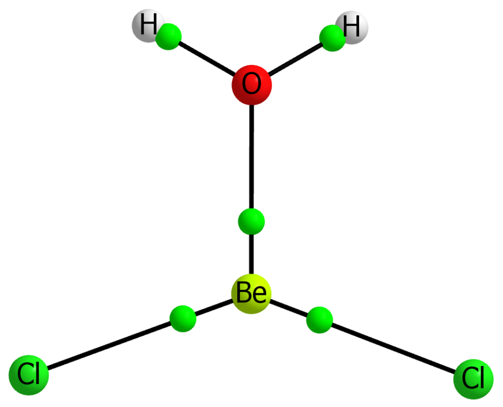
</td><td>Be
X,1,1.
Cl,1,r1,2,a1
Cl,1,r1,2,a1,3,180.,0
O,1,r2,3,a1,2,0.,0
H,5,r4,1,a4,3,d4,0
H,5,r4,1,a4,4,-d4,0

r1=1.87217475
r2=1.64178731
r4=0.9648764
a1=110.48676626
a4=120.15816917
d4=16.35346049</td></tr>
<tr><td>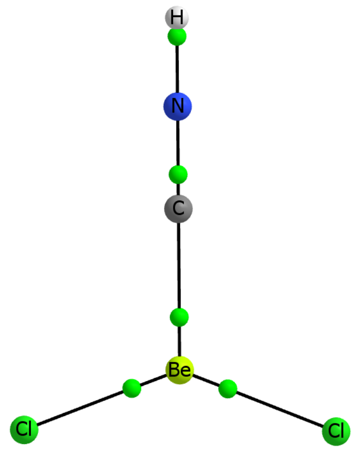
</td><td>Be
X,1,1.
Cl,1,r1,2,a1
Cl,1,r1,2,a1,3,180.,0
C,1,r2,3,a1,2,0.,0
N,1,r3,3,a1,2,0.,0
H,1,r4,3,a1,2,0.,0

r1=1.86972985
r2=1.81752714
r3=2.97136244
r4=3.9704857
a1=111.4380413</td></tr>
</table>

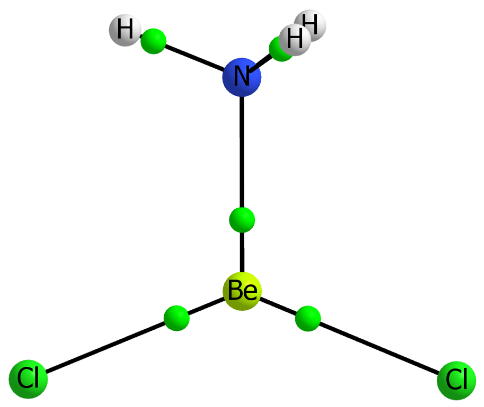

Be
X,1,1.
Cl,1,r1,2,a1
Cl,1,r1,2,a1,3,180.,0
N,1,r2,3,a1,2,0.,0
H,5,r3,1,a3,3,0.,0
H,5,r4,1,a4,3,d4,0
H,5,r4,1,a4,3,-d4,0

r1=1.87966239
r2=1.73906264
r3=1.01809737
r4=1.01882542
a1=112.56398
a3=111.8729243
a4=110.89414617
d4=121.10556264

Table S3. Electron density (au) at the BCP between the Be atom and the Lewis base.

LB	A:LB	B:LB	C:LB	D:LB	CO_3Be:LB	SO_4Be:LB	Cl_2Be:LB
N_2	0.0634	0.0633	0.0633	0.0626	0.0585	0.0583	0.0379
CO	0.0699	0.0705	0.0712	0.0706	0.0612	0.0607	0.0507
SH_2	0.0486	0.0481	0.0464	0.0459	0.0503	0.0519	0.0383
PH_3	0.0534	0.0527	0.0520	0.0515	0.0517	0.0532	0.0412
NCH	0.0735	0.0729	0.0721	0.0716	0.0720	0.0730	0.0616
OH_2	0.0711	0.0694	0.0666	0.0663	0.0774	0.0795	0.0695
CNH	0.0728	0.0730	0.0732	0.0727	0.0678	0.0685	0.0612
NH_3	0.0744	0.0731	0.0714	0.0711	0.0771	0.0792	0.0702

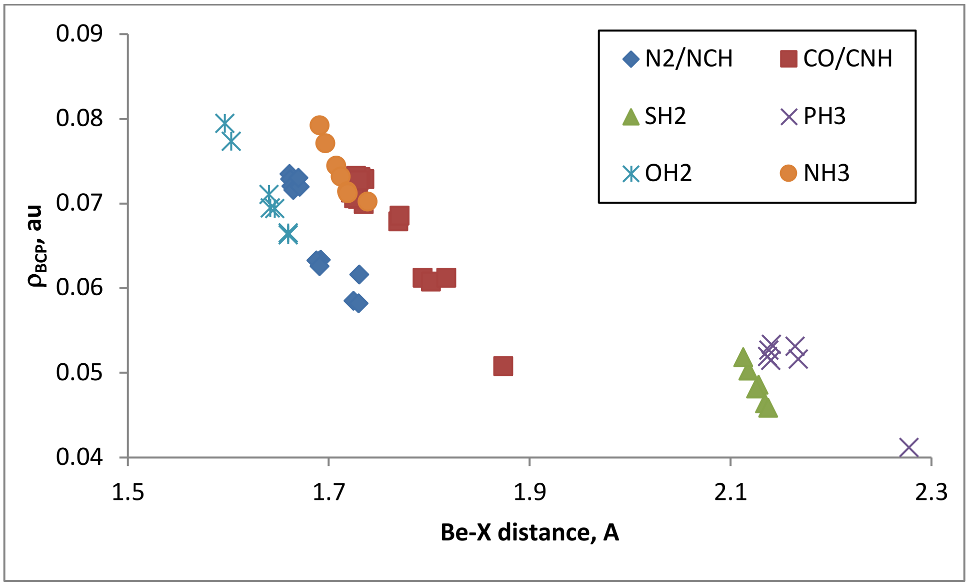

a

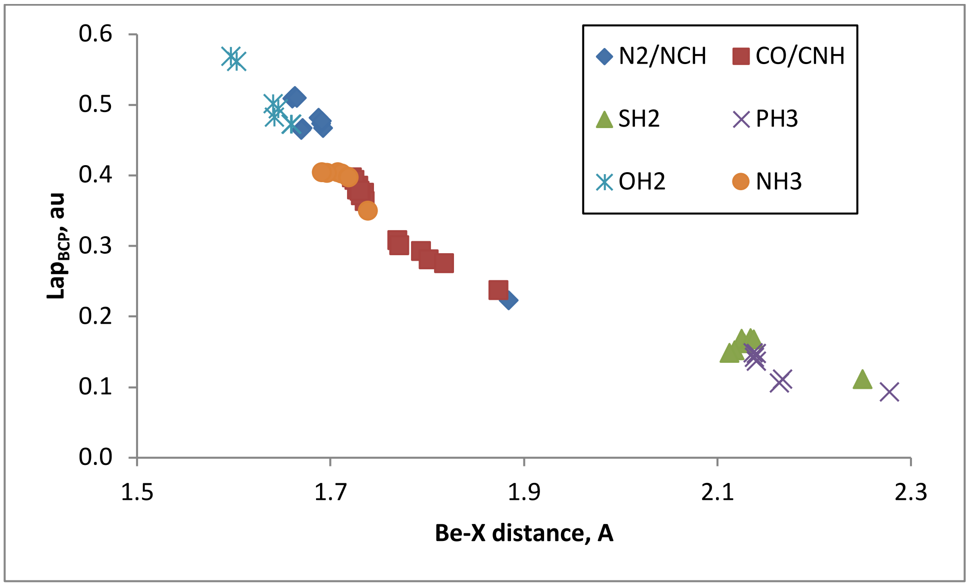

b

Fig. S4. Electron density properties (ρ_{BCP} and $\nabla^2\rho_{BCP}$) at the intermolecular BCPs (au) vs. the interatomic distance (Å).

Table S4. LMOEDA energy terms (kJ mol^{-1})

**Complexes with A**	N_2	CO	SH_2	PH_3	NCH	OH_2	CNH	NH_3
Electrostatic energy	-99.3	-136.9	-139.2	-157.0	-186.5	-213.3	-208.7	-274.6
Exchange energy	-61.2	-75.7	-71.1	-86.2	-73.1	-64.1	-82.6	-81.9
Repulsion energy	231.1	273.3	242.5	279.1	271.4	245.8	292.6	302.7
Polarization energy	-147.2	-176.6	-132.9	-154.5	-167.1	-142.3	-177.4	-151.6
Dispersion energy	-26.7	-27.9	-28.2	-28.4	-29.4	-29.2	-28.7	-31.5
Total LMOEDA energy	-103.4	-143.8	-128.9	-147.0	-184.7	-203.0	-204.8	-236.9

**Complexes with B**	N_2	CO	SH_2	PH_3	NCH	OH_2	CNH	NH_3
Electrostatic energy	-97.5	-133.8	-135.6	-151.4	-179.7	-205.1	-202.8	-266.7
Exchange energy	-60.9	-75.0	-71.1	-85.8	-72.7	-62.3	-83.0	-80.5
Repulsion energy	228.8	269.1	240.2	275.1	268.3	239.1	291.8	296.9
Polarization energy	-144.4	-173.8	-127.9	-148.7	-161.9	-136.4	-174.2	-145.4
Dispersion energy	-27.2	-28.7	-28.3	-28.6	-29.7	-29.2	-29.2	-31.6
Total LMOEDA energy	-101.2	-142.2	-122.7	-139.4	-175.7	-193.8	-197.4	-227.3

**Complexes with C**	N_2	CO	SH_2	PH_3	NCH	OH_2	CNH	NH_3
Electrostatic energy	-94.7	-131.2	-127.4	-144.3	-171.5	-192.1	-195.9	-255.7
Exchange energy	-59.8	-74.8	-68.5	-84.4	-71.0	-58.7	-82.5	-77.2
Repulsion energy	224.2	266.6	229.6	267.1	261.3	226.7	288.4	284.9
Polarization energy	-141.9	-172.8	-119.7	-142.2	-156.3	-128.0	-171.1	-137.7
Dispersion energy	-27.5	-29.2	-28.1	-28.7	-29.7	-29.0	-29.6	-31.4
Total LMOEDA energy	-99.7	-141.4	-114.2	-132.4	-167.2	-181.0	-190.7	-217.0

**Complexes with D**	N_2	CO	SH_2	PH_3	NCH	OH_2	CNH	NH_3
Electrostatic energy	-94.6	-130.8	-125.9	-142.8	-170.0	-190.0	-194.1	-254.3
Exchange energy	-60.1	-75.1	-68.4	-84.3	-71.3	-58.7	-82.8	-77.6
Repulsion energy	225.1	267.4	229.6	267.3	262.3	226.8	289.2	285.9
Polarization energy	-139.8	-170.3	-117.8	-139.9	-154.6	-127.3	-169.0	-136.4
Dispersion energy	-27.4	-29.2	-28.0	-28.6	-29.7	-28.9	-29.6	-31.4
Total LMOEDA energy	-96.9	-137.9	-110.5	-128.4	-163.3	-178.1	-186.2	-213.8

**Complexes with SO_4Be**	N_2	CO	SH_2	PH_3	NCH	OH_2	CNH	NH_3
Electrostatic energy	-81.5	-111.3	-130.7	-145.6	-186.3	-220.1	-201.4	-286.2
Exchange energy	-36.1	-40.8	-48.7	-54.6	-49.0	-46.3	-51.5	-59.3
Repulsion energy	154.3	169.5	187.5	201.0	207.4	203.6	210.1	251.8
Polarization energy	-128.0	-142.6	-137.5	-149.0	-157.8	-151.2	-157.2	-151.3
Dispersion energy	-21.2	-21.3	-25.5	-25.0	-26.5	-28.5	-24.7	-30.0
Total LMOEDA energy	-112.6	-146.6	-155.0	-173.1	-212.4	-242.4	-224.7	-275.0

**Complexes with CO_3Be**	N_2	CO	SH_2	PH_3	NCH	OH_2	CNH	NH_3
Electrostatic energy	-82.6	-112.1	-129.2	-141.9	-182.5	-214.9	-197.7	-279.2

Exchange energy	-38.7	-43.8	-50.5	-56.2	-51.2	-47.7	-54.0	-61.0
Repulsion energy	161.0	177.5	189.3	202.5	210.9	203.8	215.3	252.2
Polarization energy	-127.8	-143.3	-129.7	-141.4	-152.9	-144.3	-153.6	-143.5
Dispersion energy	-21.5	-21.6	-25.1	-24.6	-26.5	-28.2	-24.8	-29.8
Total LMOEDA energy	-109.6	-143.3	-145.3	-161.6	-202.1	-231.3	-214.8	-261.3

**Complexes with Cl_2Be**	N_2	CO	SH_2	PH_3	NCH	OH_2	CNH	NH_3
Electrostatic energy	-94.7	-135.2	-139.2	-149.9	-193.6	-242.1	-208.2	-291.3
Exchange energy	-69.8	-90.3	-85.8	-95.9	-100.8	-104.8	-106.4	-117.6
Repulsion energy	251.8	322.6	290.6	318.4	356.4	370.7	371.1	411.0
Polarization energy	-101.2	-144.2	-111.2	-127.2	-155.3	-155.7	-165.2	-157.4
Dispersion energy	-23.8	-26.8	-27.7	-27.7	-30.1	-33.1	-29.3	-34.0
Total LMOEDA energy	-37.7	-73.9	-73.2	-82.3	-123.3	-165.0	-137.9	-189.2

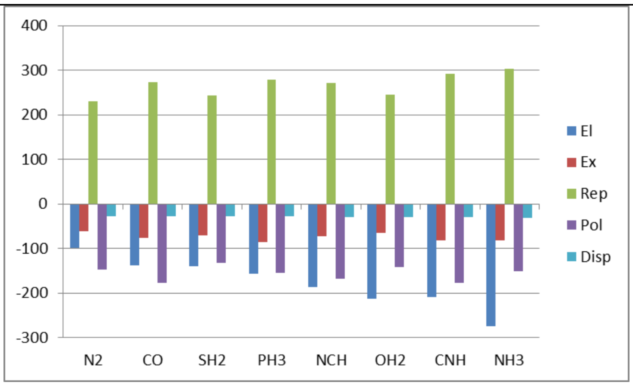

Complexes with A

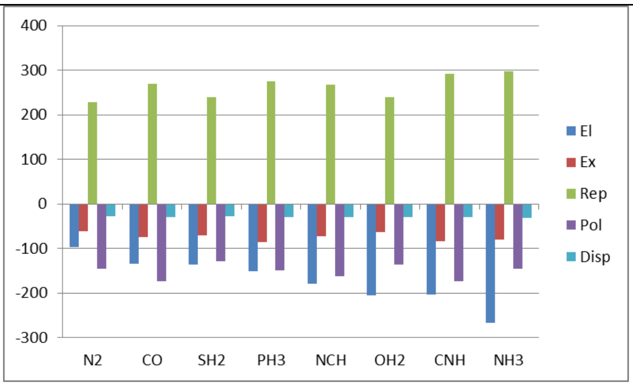

Complexes with B

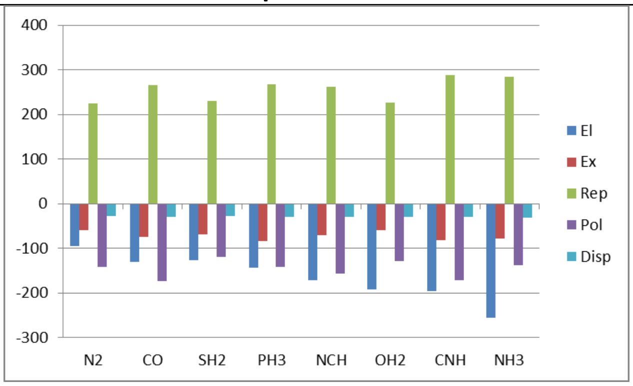

Complexes with C

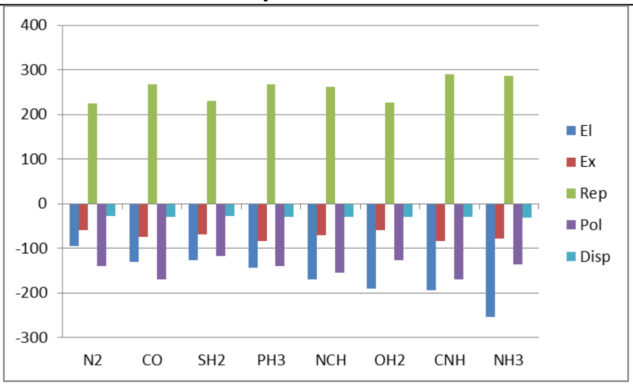

Complexes with D

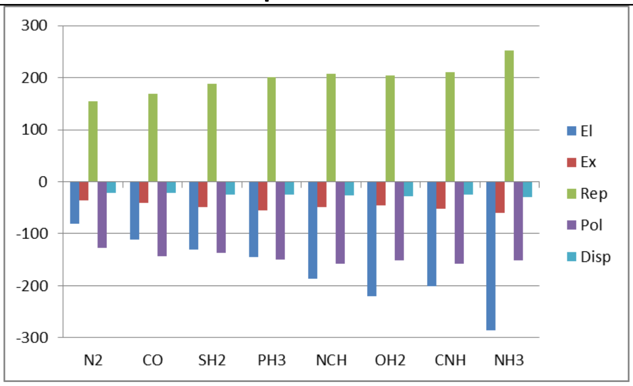

Complexes with SO_4Be

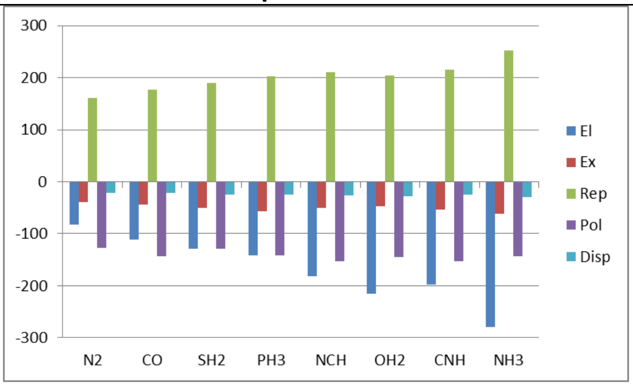

Complexes with CO_3Be

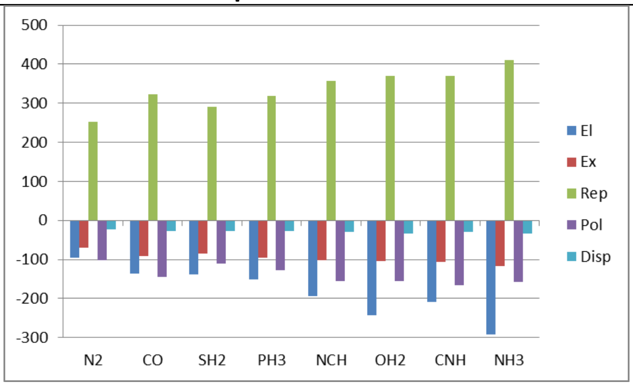

Complexes with Cl_2Be

Fig. S5. Graphical representation of the LMOEDA energy terms (kJ mol^{-1})

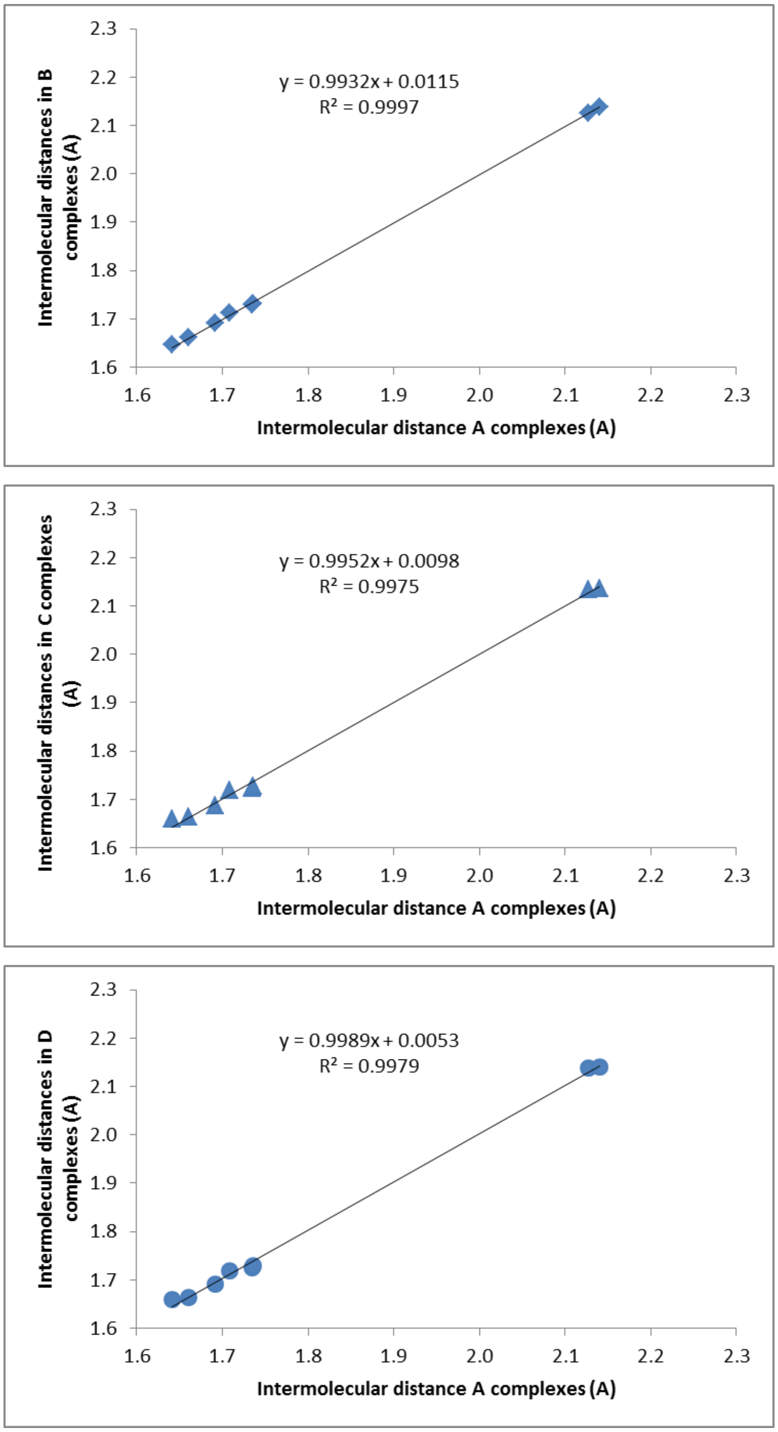

Fig. S6. Intermolecular distances in A complexes vs. B-D complexes (Å)

Table S5. Dissociation ΔG energies (kJ mol^{-1})

LB	A:LB	B:LB	C:LB	D:LB	CO_3Be:LB	SO_4Be:LB	Cl_2Be:LB
N_2	72.3	69.3	44.4	18.3	56.8	66.5	-25.3
CO	102.3	100.1	76.0	49.2	78.6	88.9	-9.0
SH_2	121.5	113.9	85.3	58.0	109.8	123.7	12.3
PH_3	132.0	129.7	100.6	71.9	118.7	133.3	15.9
NCH	151.3	142.2	111.4	83.8	145.2	160.9	35.8
OH_2	152.7	143.4	115.9	83.6	150.8	166.3	42.5
CNH	165.0	157.5	128.1	100.0	159.5	173.9	62.7
NH_3	201.8	191.5	160.3	133.3	208.1	223.6	95.8

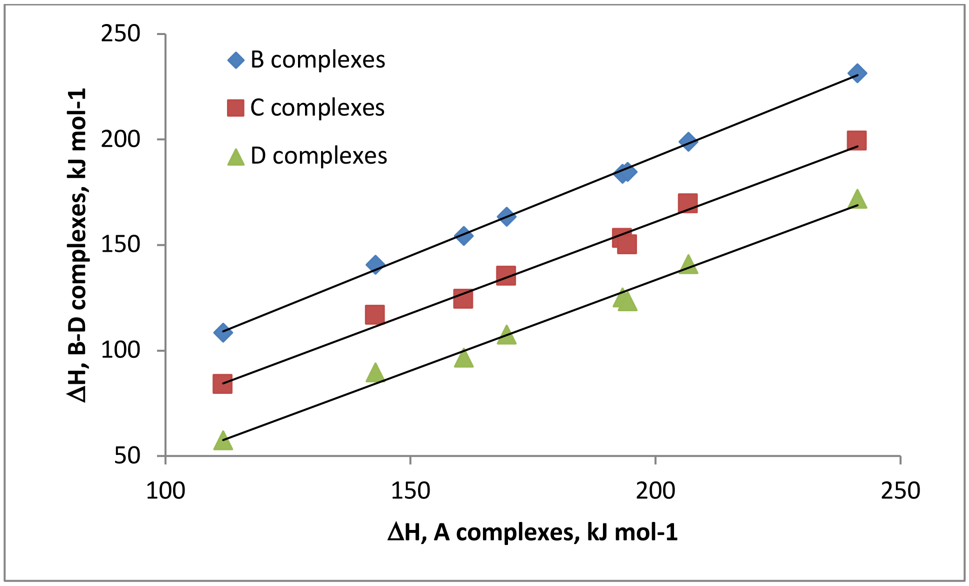

Fig. S7. Dissociation enthalpies of A complexes vs. B-D complexes (kJ mol^{-1})
